# System Performance Analysis for an Energy Harvesting IoT System Using a DF/AF UAV-Enabled Relay with Downlink NOMA under Nakagami-*m* Fading

**DOI:** 10.3390/s21010285

**Published:** 2021-01-04

**Authors:** Anh-Nhat Nguyen, Van Nhan Vo, Chakchai So-In, Dac-Binh Ha

**Affiliations:** 1 Applied Network Technology (ANT) Laboratory, Department of Computer Science, Faculty of Science, Khon Kaen University, Khon Kaen 40002, Thailand; nguyenanhnhat@kkumail.com; 2 Faculty of Information Technology, Duy Tan University, Da Nang 550000, Vietnam; vonhanvan@dtu.edu.vn; 3 Institute of Research and Development, Duy Tan University, Da Nang 550000, Vietnam; 4 Faculty of Electrical-Electronic Engineering, Duy Tan University, Da Nang 550000, Vietnam; hadacbinh@duytan.edu.vn

**Keywords:** internet of things, unmanned aerial vehicles, energy harvesting, nonorthogonal multiple access, time switching, adaptive power splitting

## Abstract

This paper investigates system performance in the Internet of Things (IoT) with an energy harvesting (EH) unmanned aerial vehicle (UAV)-enabled relay under Nakagami-*m* fading, where the time switching (TS) and adaptive power splitting (APS) protocols are applied for the UAV. Our proposed system model consists of a base station (BS), two IoT device (ID) clusters (i.e., a far cluster and a near cluster), and a multiantenna UAV-enabled relay (UR). We adopt a UR-aided TS and APS (U-TSAPS) protocol, in which the UR can dynamically optimize the respective power splitting ratio (PSR) according to the channel conditions. To improve the throughput, the nonorthogonal multiple access (NOMA) technique is applied in the transmission of both hops (i.e., from the BS to the UR and from the UR to the ID clusters). The U-TSAPS protocol is divided into two phases. In the first phase, the BS transmits a signal to the UR. The UR then splits the received signal into two streams for information processing and EH using the APS scheme. In the second phase, the selected antenna of the UR forwards the received signal to the best far ID (BFID) in the far cluster and the best near ID (BNID) in the near cluster using the decode-and-forward (DF) or amplify-and-forward (AF) NOMA scheme. We derive closed-form expressions for the outage probabilities (OPs) at the BFID and BNID with the APS ratio under imperfect channel state information (ICSI) to evaluate the system performance. Based on these derivations, the throughputs of the considered system are also evaluated. Moreover, we propose an algorithm for determining the nearly optimal EH time for the system to minimize the OP. In addition, Monte Carlo simulation results are presented to confirm the accuracy of our analysis based on simulations of the system performance under various system parameters, such as the EH time, the height and position of the UR, the number of UR antennas, and the number of IDs in each cluster.

## 1. Introduction

In recent years, the Internet of Things (IoT) paradigm has undergone incredible growth and received extensive attention [1,2,3,4,5]. The IoT requires a large number of sensors deployed throughout a huge space, including in remote areas and areas that are inaccessible to humans [6,7]. Under such circumstances, collecting sensor data becomes a challenging task. Consequently, unmanned aerial vehicles (UAVs) have emerged as a practical solution to such problems [8,9,10].

UAVs offer advantageous performance because of their maneuverability, which allows the position of a UAV to be automatically adjusted to best suit the current communication needs [11]. For example, in [12], the authors considered a UAV-enabled relay (UR) network with a UAV acting as a decode-and-forward (DF) relay to extend the coverage of the BS. Song et al. studied the use of a UAV as an aerial amplify-and-forward (AF) relay to receive information being transmitted from the BS to an IoT device (ID) and considered how to maximize the energy efficiency of a UAV-supported IoT system [13].

A UAV is a highly mobile device, but one of its drawbacks is the requirement for a strong power source to be able to sustain its flight time and communication processes [14]. Recently, radio frequency (RF) EH has emerged as a promising solution for prolonging the lifetime of low-power devices due to its constant energy production capabilities [15,16]. Some studies have explored UR-assisted communication with RF EH. For example, Hua et al. analyzed an AF UR network in which the UR harvests energy using a power splitting (PS) protocol and derives multiple parameters through joint optimization to maximize the system throughput [17]. The outage probability (OP) of a DF UR with PS and time switching (TS) protocols was analyzed in [18] under the assumption of a perfect UR-to-destination channel, with the BS-to-UR channel modeled as a Nakagami-*m* fading channel with interference at the destination. However, an equal time switching ratio (TSR) for data transfer was assumed, and the PSR was not optimized.

To further enhance the system capacity and provide an enhanced experience for the IDs, especially for IDs at a cell edge, nonorthogonal multiple access (NOMA) has recently been proposed as a promising solution for 5G wireless networks [19,20,21]. Furthermore, the NOMA technique can also be used for performance enhancement in UAV-enabled wireless networks [22,23]. For example, in [24], a power allocation scheme was proposed to maximize the sum rate in NOMA. The results showed that the proposed scheme achieved a better sum rate than the classical system. However, UAVs communicate with ground users through air links, and the channel state information (CSI) is not perfectly perceived in practice due to estimation errors and finite data feedback [25], as discussed in regard to the resource allocation problem for cellular networks operating under the NOMA scheme with imperfect channel state information (ICSI). Therefore, in [26], the authors maximized the energy efficiency in a UAV-enabled NOMA downlink network while considering ICSI between the UAV and the IDs.

In addition, for an RF EH UR operating based on a PS protocol, the received signal is divided into two parts throughout the whole receiving time. One part is fed to the EH circuitry, while the other part is fed to the information processing circuitry. Currently, PS protocols can be divided into two categories: fixed power splitting (FPS) protocols and adaptive power splitting (APS) protocols. In an FPS protocol, the PSR is constant across all receiving times. However, the system performance depends on parameters such as the PSR. Therefore, to achieve optimal performance, this parameter needs to be adaptive.

Motivated by the above discussion, we study the performance of a UR IoT communication network using NOMA with ICSI under Nakagami-*m* fading channels. Note that, the Nakagami-*m* distribution is a general case that encompasses three distributions as special cases. Thus, the analysis for Nakagami-*m* fading is equally applicable in any of the corresponding fading environments (m<1 for Hoyt, m=1 for Rayleigh, and m>1 for Rician) [27]. To save the electric power of the UAV’s battery and enhance its flight endurance, we apply the RF EH technique for the UAV, where the energy harvested from the BS is used for the transmit power of the UR [28]. Specifically, we adopt the UR-aided TS and APS (U-TSAPS) protocol, in which the UR can adjust the APS ratio in accordance with the channel conditions. Moreover, the DF/AF schemes are adopted at the UR. The main contributions of this paper are summarized as follows:

- We investigate a UR-assisted IoT communication network using RF EH and downlink NOMA with ICSI under Nakagami-*m* fading channels.
- We derive an APS ratio that maximizes the channel capacity of the system. The channel capacity maximization is achieved by maximizing the signal-to-interference-plus-noise ratio (SINR) of the system.
- We also derive closed-form expressions for the system OPs for the DF and AF schemes, considering the APS ratio. In addition, the system throughputs are obtained for both the DF and AF schemes.
- We propose an algorithm for finding the nearly optimal EH time for the system.

The remainder of this paper is organized as follows. In Section 2, related work on UR-assisted IoT communication networks using RF EH and NOMA is presented. In Section 3, the system model, the communication protocol, and the APS protocol for DF and AF are introduced. In Section 4, the OPs for the DF and AF schemes is analyzed. In Section 5, numerical results are presented and discussed. Finally, conclusions and future work are presented in Section 6.

## 2. Related Work

In this section, we briefly summarize the related work concerning UR-assisted communication networks.

The UAV-enabled relay (UR) concept, in which UAVs are deployed to assist in communication between terrestrial nodes, has been investigated in the literature under various relaying schemes, including the DF and AF schemes [29,30,31]. In [29], it was shown that the communication throughput achievable with a DF UR can be significantly improved compared with that achievable using a conventional DF relay by allowing the UR to transmit when it is flying closer to the destination and receive when it is flying closer to the source. In contrast to DF relaying, the work presented in [30] studied the use of an AF UR to minimize the communication OP and maximize the communication throughput. Furthermore, Chen et al. analyzed the outage performance of DF and AF URs under Nakagami-*m* fading channels. However, the above studies on URs were performed without considering EH, mostly focusing on analyzing the OP and throughput of the system.

Later, RF EH technology was introduced to alleviate the energy consumption problem for UAV equipment [32,33,34]. For example, Yang et al. studied the performance of UR networks in urban environments. The OP of an AF UR with a TS protocol was analyzed by considering Rician fading for the channel from the BS to the UR and shadowed Rayleigh fading for the channel from the UR to the destination [32]. In addition, Yin et al. studied the throughput maximization problem with a focus on UAV-assisted wireless communication, considering a communication system with one pair of a BS and an ID, in which a UAV serves as an aerial relay based on an AF scheme and harvests energy using a PS protocol [33]. However, the studies above on RF EH URs focused only on analyzing the OP and throughput of a system with an equal TSR for data transfer and a constant PSR across all receiving times. In contrast, Kim et al. considered a DF UR network in which the UR performs EH and information decoding simultaneously with a PS protocol, and they adapted the PS ratio to minimize the OP for transferring data from the source to the destination. To evaluate the performance of this system, the OP with the proposed APS ratio was analyzed under a generalized UR channel model with Nakagami-*m* fading [34]. However, this work focused on a simplified system (i.e., a source, a UR and a destination).

On the other hand, UAV-enabled communication and NOMA can be combined in a hybrid network to achieve both superior spectral efficiency and ubiquitous coverage [35,36]. Wang et al. considered a DF UR system based on downlink NOMA, in which the UR forwards information from the BS to multiple access points. They presented a joint design for the power allocation for NOMA at the UR to minimize the maximum OP among all links [35]. In addition, Zaidi et al. proposed a network in which ground users and a UR use NOMA, with the DF UR playing the role of extending the coverage of the source. The performance of the proposed model was evaluated based on the OPs with different levels of transmit power and fading environments. Moreover, the authors compared the system throughput of the proposed system with that of an orthogonal multiple access (OMA)-based network and showed that their proposed network was superior in performance [36]. However, EH at the UR to further enhance the system performance was not considered in the studies discussed above.

Note that the above works assumed perfect CSI to be feasible at the receiver; however, perfect CSI is difficult to obtain because of channel estimation errors [26]. Thus, considering ICSI in wireless communication systems is essential to investigate systems that are representative of real-world applications. In [37], the performance of a DF UR-assisted NOMA network over Rician fading channels was studied. The authors derived approximate closed-form expressions for the OPs for a far user and a near user under ICSI conditions. Additionally, system throughput is evaluated and discussed. However, although the impact of ICSI on the system performance was analyzed, EH at the UR to enhance the system performance was not investigated.

Based on the above review, URs that apply the RF EH and NOMA techniques simultaneously have not been studied extensively in recent works. Thus, in this paper, we focus on APS for an RF EH IoT system using DF/AF UR downlink NOMA with ICSI under Nakagami-*m* fading.

## 3. System and Channel Model

### 3.1. System Model

**Figure 1:**
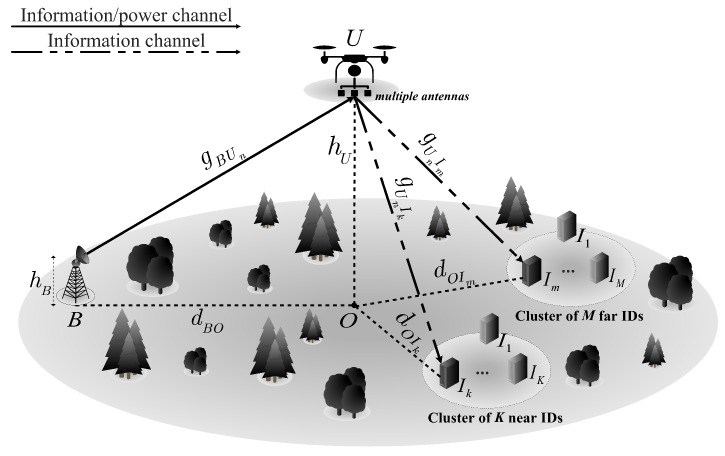
System model for an EH UR downlink NOMA system in the IoT context.

In this paper, we consider the downlink NOMA UR assisted IoT system depicted in Figure 1, where the system model consists of a BS *B*, two clusters (i.e., a far cluster with *M* IDs and a near cluster with *K* IDs), and single energy-limited UR *U* that uses the DF or AF scheme to send the information it collects to the IDs. All nodes are operated in half-duplex mode. We assume that the BS and IDs are single-antenna devices and that the UR is equipped with *N* antennas. It is noted that the UR equipped with multiple antennas can provide improved connectivity performance and extend the range of communication [38]. However, the use of multiple antennas also increases the impact of interference on the UR. Therefore, to reduce the effect of interference on the UR, the BS is deployed with a single antenna by adopting the approach presented in [39,40,41]. There are no direct links between *B* and the IDs due to the presence of obstacles. For clarity, we define the notations adopted throughout the remainder of this paper in Table 1.

We assume that the channel coefficients $g_{XY}$ are independent and identically distributed (i.i.d) and modeled as Nakagami-*m* fading channels [42,43] with fading parameters $m_{XY}$, $E\left\{{\left|g_{XY}\right|}^{2}\right\}=\Omega_{XY}$, and ${\left|g_{XY}\right|}^{2}$ is a random variable (RV) [19], where $XY\in \left\{BU_{n},U_{n}I_{i}\right\}$. In practice, it is not feasible to obtain perfect CSI for a wireless network due to channel estimation errors. Therefore, the channel coefficients can be modeled as [21,44]

(1) $$g_{XY}={\hat{g}}_{XY}+e_{XY},$$

where ${\hat{g}}_{XY}$ represents the estimated channel coefficient and $e_{XY}∼CN\left(0,E_{XY}\right)$ denotes the channel estimation error, which can be approximated as a Gaussian RV [20]. In addition, ${\hat{\Omega }}_{XY}=\Omega_{XY}-E_{XY}$ can be obtained by assuming that ${\hat{\Omega }}_{XY}$ is statistically independent of $e_{XY}$. Let $\mu_{XY}=E_{XY}/\Omega_{XY}\;$ denote the relative channel estimation error; we have $E_{XY}=\mu_{XY}{d_{XY}}^{-\sigma }$ and ${\hat{\Omega }}_{XY}=\left(1-\mu_{XY}\right){d_{XY}}^{-\sigma }$ [45]. Accordingly, the cumulative distribution function (CDF) and probability density function (PDF) of the channel gain ${\left|{\hat{g}}_{XY}\right|}^{2}$ are given as follows [46]:

(2) $$\begin{array}{cc} F_{{\left|{\hat{g}}_{XY}\right|}^{2}}\left(y\right) & =1-e^{-\frac{ym_{XY}}{{\hat{\Omega }}_{XY}}}{\sum_{s=0}^{m_{XY}-1}\frac{1}{s!}\left(\frac{ym_{XY}}{{\hat{\Omega }}_{XY}}\right)}^{s}, \end{array}$$

(3) $$\begin{array}{cc} f_{{\left|{\hat{g}}_{XY}\right|}^{2}}\left(y\right) & =\frac{y^{m_{XY}-1}}{\left(m_{XY}-1\right)!}{\left(\frac{m_{XY}}{{\hat{\Omega }}_{XY}}\right)}^{m_{XY}}e^{-\frac{ym_{XY}}{{\hat{\Omega }}_{XY}}}. \end{array}$$

### 3.2. Communication Protocol

**Figure 2:**
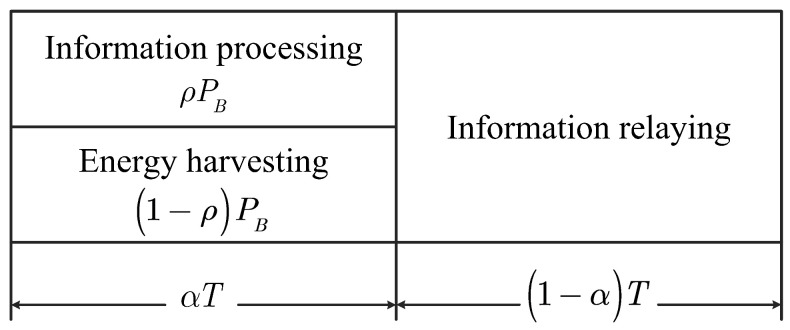
The U-TSAPS protocol. The time block *T* is used for both the information processing and EH phase and the information relaying phase.

In the considered system, we apply a U-TSAPS communication protocol that is divided into two phases, as illustrated in Figure 2. This communication protocol is described as follows:

- In the first phase, *B* transmits information $x_{B}=\sqrt{a_{k}}x_{k}+\sqrt{a_{m}}x_{m}$ to $U_{n}$ in accordance with the NOMA principle within a length of time αT, where $x_{m}$ and $x_{k}$ are the signals to be received by $I_{m}$ and $I_{k}$ and $a_{m}$ and $a_{k}$ are power allocation coefficients that satisfy the conditions $a_{m}+a_{k}=1$ and $a_{m}>a_{k}$ [20]. As described in [47], at $U_{n}$, the received power is divided into two streams, with $\rho P_{B}$ for information processing and $\left(1-\rho \right)P_{B}$ for EH (the APS ratio in Section III.C). Thus, the signal received at $U_{n}$ is written as follows:
  (4) $$\begin{array}{c} z_{U_{n}}=\sqrt{\rho P_{B}}\left(\sqrt{a_{k}}x_{k}+\sqrt{a_{m}}x_{m}\right)g_{BU_{n}}+n_{U_{n}}, \end{array}$$
  where $g_{BU_{n}}={\hat{g}}_{BU_{n}}+e_{BU_{n}}$, $P_{B}$ is the transmit power of *B*, and $n_{U_{n}}∼CN\left(0,N_{0}\right)$ is additive white Gaussian noise (AWGN) [21,48].
  Thus, the energy harvested at $U_{n}$ can be expressed as follows [49,50,51]:
  (5) $$\begin{array}{c} E_{U_{n}}=\eta \alpha \left(1-\rho \right)P_{B}\left({\left|{\hat{g}}_{BU_{n}}\right|}^{2}+E_{BU_{n}}\right)T, \end{array}$$
  where η is the EH efficiency coefficient, which depends on the rectification (0<η<1). Here, we assume that the EH efficiency coefficient is the same for all antennas of the UR.
  The antenna $U_{n}$ of the UR first decodes the message for $I_{m}$ (i.e., $x_{m}$) by treating the message for $I_{k}$ (i.e., $x_{k}$) as noise (because the power allocation coefficient for $I_{m}$ is higher than that for $I_{k}$). $U_{n}$ then cancels out the message $x_{m}$ using successive interference cancellation (SIC) to obtain the message $x_{k}$. Here, we assume that $U_{n}$ can decode $x_{k}$ successfully by adopting the method proposed in [52,53,54]. Therefore, the received signal-to-interference-plus-noise ratios (SINRs) at $U_{n}$ for detecting $x_{m}$ and $x_{k}$ are expressed as follows:
  (6) $$\begin{array}{cc} \; & \gamma_{BU_{n}}^{x_{m}}=\frac{\rho P_{B}a_{m}{\left|{\hat{g}}_{BU_{n}}\right|}^{2}}{\rho P_{B}a_{k}{\left|{\hat{g}}_{BU_{n}}\right|}^{2}+\rho P_{B}E_{BU_{n}}+N_{0}}, \end{array}$$
  (7) $$\begin{array}{cc} \; & \gamma_{BU_{n}}^{x_{k}}=\frac{\rho P_{B}a_{k}{\left|{\hat{g}}_{BU_{n}}\right|}^{2}}{\rho P_{B}E_{BU_{n}}+N_{0}}, \end{array}$$
- In the second phase, the transmit power at the *n*-th antenna during the remaining time $\left(1-\alpha \right)T$ is expressed as
  (8) $$\begin{array}{cc} P_{U_{n}} & =\frac{E_{U_{n}}}{\left(1-\alpha \right)T}=\upsilon \left(1-\rho \right)P_{B}\left({\left|{\hat{g}}_{BU_{n}}\right|}^{2}+E_{BU_{n}}\right), \end{array}$$
  where $\upsilon =\frac{\eta \alpha }{\left(1-\alpha \right)}$.
  Furthermore, UAV $U_{n}$ utilizes either DF or AF scheme to perform relaying transmission.
  (1) DF scheme
    For the DF scheme, UR first decodes the received superimposed messages from *B* and then re-encodes and forwards them to the IDs. Then, the received signal at $I_{i}$ is given by
    (9) $$\begin{array}{c} z_{I_{i}}^{DF}=\sqrt{P_{U_{n}}}\left(\sqrt{a_{k}}x_{k}+\sqrt{a_{m}}x_{m}\right)g_{U_{n}I_{i}}+n_{I_{i}}, \end{array}$$
    where $g_{U_{n}I_{i}}={\hat{g}}_{U_{n}I_{i}}+e_{U_{n}I_{i}}$ and $n_{I_{i}}∼CN\left(0,N_{0}\right)$ is the AWGN at $I_{i}$. Thus, the SINRs for detecting $x_{m}$ and $x_{k}$ transmitted from $U_{n}$ at $I_{m}$ and $I_{k}$ are expressed as
    (10) $$\begin{array}{cc} \; & \gamma_{U_{n}I_{m}}^{DF}=\frac{P_{U_{n}}a_{m}{\left|{\hat{g}}_{U_{n}I_{m}}\right|}^{2}}{P_{U_{n}}a_{k}{\left|{\hat{g}}_{U_{n}I_{m}}\right|}^{2}+P_{U_{n}}E_{U_{n}I_{m}}+N_{0}}, \end{array}$$
    (11) $$\begin{array}{cc} \; & \gamma_{U_{n}I_{k}}^{DF}=\frac{P_{U_{n}}a_{k}{\left|{\hat{g}}_{U_{n}I_{k}}\right|}^{2}}{\rho \gamma_{B}E_{U_{n}I_{k}}+N_{0}}. \end{array}$$
  (2) AF scheme
    For the AF scheme, $U_{n}$ transmits $z_{U_{n}}$ to all IDs after multiplying it by an amplifying factor $G_{n}$. To satisfy the output power constraint at $U_{n}$, it is required that $E\left\{{\left|G_{n}z_{U_{n}}\right|}^{2}\right\}=P_{U_{n}}$, where $P_{U_{n}}$ is given in (8). Here, the amplification factor is approximated by assuming a high signal-to-noise ratio (SNR) from *B* to $U_{n}$ [55,56]. Thus, $G_{n}$ is given by
    (12) $$\begin{array}{cc} G_{n} & =\sqrt{\frac{\upsilon \left(1-\rho \right)P_{B}\left({\left|{\hat{g}}_{BU_{n}}\right|}^{2}+E_{BU_{n}}\right)}{\rho \left[P_{B}\left({\left|{\hat{g}}_{BU_{n}}\right|}^{2}+E_{BU_{n}}\right)+N_{0}\right]}}≃\sqrt{\frac{\upsilon \left(1-\rho \right)}{\rho }}. \end{array}$$
    Therefore, the signal received at $I_{i}$ is given by
    (13) $$\begin{array}{cc} z_{I_{i}}^{AF} & =G_{n}\sqrt{\rho P_{B}}\left(\sqrt{a_{k}}x_{k}+\sqrt{a_{m}}x_{m}\right)g_{BU_{n}}g_{U_{n}I_{i}}+G_{n}g_{U_{n}I_{i}}n_{BU_{n}}+n_{I_{i}}. \end{array}$$
    Thus, the SINRs for detecting $x_{m}$ and $x_{k}$ transmitted from $U_{n}$ at $I_{m}$ and $I_{k}$ are expressed as
    (14) $$\begin{array}{cc} \gamma_{e2e,m}^{AF} & =\frac{\rho a_{m}\gamma_{B}{\left|{\hat{g}}_{BU_{n}}\right|}^{2}{\left|{\hat{g}}_{U_{n}I_{m}}\right|}^{2}}{\rho \gamma_{B}\left(a_{k}{\left|{\hat{g}}_{BU_{n}}\right|}^{2}{\left|{\hat{g}}_{U_{n}I_{m}}\right|}^{2}+{\left|{\hat{g}}_{U_{n}I_{m}}\right|}^{2}E_{BU_{n}}+{\left|{\hat{g}}_{BU_{n}}\right|}^{2}E_{U_{n}I_{m}}+E_{BU_{n}}E_{U_{n}I_{m}}\right)+{\left|{\hat{g}}_{U_{n}I_{m}}\right|}^{2}+E_{U_{n}I_{m}}+\rho /\upsilon \left(1-\rho \right)\;}, \end{array}$$
    (15) $$\begin{array}{cc} \gamma_{e2e,k}^{AF} & =\frac{\rho a_{k}\gamma_{B}{\left|{\hat{g}}_{BU_{n}}\right|}^{2}{\left|{\hat{g}}_{U_{n}I_{k}}\right|}^{2}}{\rho \gamma_{B}\left({\left|{\hat{g}}_{U_{n}I_{k}}\right|}^{2}E_{BU_{n}}+{\left|{\hat{g}}_{BU_{n}}\right|}^{2}E_{U_{n}I_{k}}+E_{BU_{n}}E_{U_{n}I_{k}}\right)+{\left|{\hat{g}}_{U_{n}I_{k}}\right|}^{2}+E_{U_{n}I_{k}}+\rho /\upsilon \left(1-\rho \right)\;}, \end{array}$$
    where $\gamma_{B}=\frac{P_{B}}{N_{0}}$.

### 3.3. APS Ratio

In this subsection, we present the APS ratios ρ for the DF and AF schemes.

- DF scheme
  The PS protocol is applied in the EH process to improve the reliability of transmission. That is, as much energy is harvested from the signals as possible under the condition that the signals received at *U* can be decoded successfully.
  Let the target SINRs for $I_{m}$ and $I_{k}$ be denoted by $\gamma_{m}$ and $\gamma_{k}$, respectively. Therefore, at $U_{n}$, the received SINRs for decoding the signals must satisfy $\gamma_{BU_{n}}^{x_{m}}\geq \gamma_{m}$ and $\gamma_{BU_{n}}^{x_{k}}\geq \gamma_{k}$. To harvest as much energy as possible, we let $\gamma_{BU_{n}}^{x_{m}}=\gamma_{m}$ and $\gamma_{BU_{n}}^{x_{k}}=\gamma_{k}$, that is [57],
  (16) $$\left\{\begin{array}{cc} \frac{\rho \gamma_{B}a_{m}{\left|{\hat{g}}_{BU_{n}}\right|}^{2}}{\rho \gamma_{B}a_{k}{\left|{\hat{g}}_{BU_{n}}\right|}^{2}+\rho \gamma_{B}E_{BU_{n}}+1} & =\gamma_{m},\\ \frac{\rho \gamma_{B}a_{k}{\left|{\hat{g}}_{BU_{n}}\right|}^{2}}{\rho \gamma_{B}E_{BU_{n}}+1} & =\gamma_{k}, \end{array}\right.$$
  where $\gamma_{m}=2^{\frac{R_{m}}{1-\alpha }}-1$ and $\gamma_{k}=2^{\frac{R_{k}}{1-\alpha }}-1$. Here, $R_{m}$ and $R_{k}$ denote the target data rates for IDs in the two clusters. After some algebraic manipulations, (16) can be rewritten as follows:
  (17) $$\frac{\left(1-a_{k}-\gamma_{m}a_{k}\right)}{a_{k}}=\frac{\gamma_{m}}{\gamma_{k}}.$$
  From (17), it is easy to find that the power allocation coefficient $a_{k}$ is given by
  (18) $$\begin{array}{c} a_{k}=\frac{\gamma_{k}}{\varpi }, \end{array}$$
  where $\varpi =2^{\frac{R_{m}+R_{k}}{1-\alpha }}-1$. By substituting (18) into (16), the APS ratio $\rho_{*}^{DF}$ can be expressed as
  (19) $$\begin{array}{c} \rho_{*}^{DF}=\frac{\varpi }{\gamma_{B}\left({\left|{\hat{g}}_{BU_{n}}\right|}^{2}-\varpi E_{BU_{n}}\right)}. \end{array}$$
  Note that $0\leq \rho_{*}^{DF}\leq 1$. When $\rho_{*}^{DF}=1$, this means that all of the energy of the received signals must be used to decode information, and the relay cannot harvest any energy. Thus, $\rho_{*}^{DF}$ can be further expressed as
  (20) $$\begin{array}{c} \rho_{*}^{DF}=\min\left(1,\frac{\varpi }{\gamma_{B}\left({\left|{\hat{g}}_{BU_{n}}\right|}^{2}-\varpi E_{BU_{n}}\right)}\right). \end{array}$$

**Remark** **1.** The UR uses the DF scheme to decode the signals from the BS. The UR has to first ensure the detection of the messages from the BS; then, it can carry out EH. Therefore, the choice of the PSR in (19) is optimal [57,58,59]. Particularly, if the PSR is set as $\rho >\rho_{*}^{DF}$, too much power is directed to the EH circuit. and there is not sufficient signal power for decoding, which leads to a decoding failure. If the PSR is set as $\rho <\rho_{*}^{DF}$, too much signal power is directed to the detection circuit: the circuit needs only $\rho_{*}^{DF}P_{B}$ to guarantee correct decoding. Therefore, a choice of $\rho <\rho_{*}^{DF}$ leads to an inefficient use of the incoming signals. Thus, the optimal choice of the PSR is $\rho =\rho_{*}^{DF}$.

Based on (8) and (20), the maximal transmission power at *U* can be obtained as follows:
  (21) $$\begin{array}{cc} P_{U_{n}} & =\upsilon \gamma_{B}\left({\left|{\hat{g}}_{BU_{n}}\right|}^{2}+E_{BU_{n}}\right)-\frac{\upsilon \varpi \left({\left|{\hat{g}}_{BU_{n}}\right|}^{2}+E_{BU_{n}}\right)}{\left({\left|{\hat{g}}_{BU_{n}}\right|}^{2}-\varpi E_{BU_{n}}\right)}. \end{array}$$
Thus, based on (10), (11), and (21), the end-to-end SINRs at $I_{k}$ and $I_{m}$ can be derived
  (22) $$\begin{array}{cc} \gamma_{e2e,m}^{DF} & =\frac{\left({\left|{\hat{g}}_{BU_{n}}\right|}^{2}-\Delta \right)a_{m}{\left|{\hat{g}}_{U_{n}I_{m}}\right|}^{2}}{\left({\left|{\hat{g}}_{BU_{n}}\right|}^{2}-\Delta \right)a_{k}{\left|{\hat{g}}_{U_{n}I_{m}}\right|}^{2}+\left({\left|{\hat{g}}_{BU_{n}}\right|}^{2}-\Delta \right)E_{U_{n}I_{m}}+\left[\left({\left|{\hat{g}}_{BU_{n}}\right|}^{2}-\varpi E_{BU_{n}}\right)/\upsilon \gamma_{B}\left({\left|{\hat{g}}_{BU_{n}}\right|}^{2}+E_{BU_{n}}\right)\;\right]}, \end{array}$$
  (23) $$\begin{array}{cc} \gamma_{e2e,k}^{DF} & =\frac{\left({\left|{\hat{g}}_{BU_{n}}\right|}^{2}-\Delta \right)a_{k}{\left|{\hat{g}}_{U_{n}I_{k}}\right|}^{2}}{\left({\left|{\hat{g}}_{BU_{n}}\right|}^{2}-\Delta \right)E_{U_{n}I_{k}}+\left[\left({\left|{\hat{g}}_{BU_{n}}\right|}^{2}-\varpi E_{BU_{n}}\right)/\upsilon \gamma_{B}\left({\left|{\hat{g}}_{BU_{n}}\right|}^{2}+E_{BU_{n}}\right)\;\right]}, \end{array}$$
  where $\Delta =\frac{\left(1+\gamma_{B}E_{BU_{n}}\right)\varpi }{\gamma_{B}}$.

- *AF scheme*
  The goal of this subsection is to identify the APS ratio $\rho_{*,i}^{AF}$ that maximizes the SINRs:
  (24) $$\begin{array}{c} \rho_{*,i}^{AF}=\arg\max_{0\leq \rho \leq 1}\;\left(\gamma_{e2e,i}^{AF}\right), \end{array}$$
  where $\gamma_{e2e,i}^{AF}\in \left\{\gamma_{e2e,k}^{AF},\gamma_{e2e,m}^{AF}\right\}$. To find the value of ρ that maximizes $\gamma_{e2e,i}^{AF}$, we differentiate $\gamma_{e2e,i}^{AF}$ with respect to ρ and set it equal to zero. After some simplifications, we have the following possible roots for $\rho_{*,i}^{AF}$:
  (25) $$\begin{array}{cc} \; & \rho_{*,i}^{AF}=\frac{\sqrt{\upsilon \left({\left|{\hat{g}}_{U_{n}I_{i}}\right|}^{2}+E_{U_{n}I_{i}}\right)}}{1+\sqrt{\upsilon \left({\left|{\hat{g}}_{U_{n}I_{i}}\right|}^{2}+E_{U_{n}I_{i}}\right)}}, \end{array}$$
  or
  (26) $$\begin{array}{cc} \; & \rho_{*,i}^{AF}=-\frac{\sqrt{\upsilon \left({\left|{\hat{g}}_{U_{n}I_{i}}\right|}^{2}+E_{U_{n}I_{i}}\right)}}{1-\sqrt{\upsilon \left({\left|{\hat{g}}_{U_{n}I_{i}}\right|}^{2}+E_{U_{n}I_{i}}\right)}}. \end{array}$$
  Accordingly, we choose $\rho_{*,i}^{AF}=\frac{\sqrt{\upsilon \left({\left|{\hat{g}}_{U_{n}I_{i}}\right|}^{2}+E_{U_{n}I_{i}}\right)}}{1+\sqrt{\upsilon \left({\left|{\hat{g}}_{U_{n}I_{i}}\right|}^{2}+E_{U_{n}I_{i}}\right)}}$ as the root because $\rho_{*,i}^{AF}=$$-\frac{\sqrt{\upsilon \left({\left|{\hat{g}}_{U_{n}I_{i}}\right|}^{2}+E_{U_{n}I_{i}}\right)}}{1-\sqrt{\upsilon \left({\left|{\hat{g}}_{U_{n}I_{i}}\right|}^{2}+E_{U_{n}I_{i}}\right)}}<0$.

**Remark** **2.** *The end-to-end SINR $\gamma_{e2e,i}^{AF}$ is a concave function in terms of the PSR ρ. Clearly, the second-order derivative $\frac{\partial^{2}\gamma_{e2e,i}^{AF}}{\partial \rho^{2}}$ is negative for 0≤ρ≤1 [58,60,61]. Therefore, we conclude that $\gamma_{e2e,i}^{AF}$ is a concave function of ρ for 0≤ρ≤1.*

For this AF scheme, we analyze two cases of APS ratio selection to improve the system performance.
  (1) *Case I:* In this case, the PSR dynamically varies towards achieving the maximum end-to-end SINR for the signal $x_{m}$. Therefore, the APS ratio of the system is
    (27) $$\begin{array}{c} \rho_{*}^{AF,C1}=\rho_{*,m}^{AF}=\frac{\sqrt{\upsilon \left({\left|{\hat{g}}_{U_{n}I_{m}}\right|}^{2}+E_{U_{n}I_{m}}\right)}}{1+\sqrt{\upsilon \left({\left|{\hat{g}}_{U_{n}I_{m}}\right|}^{2}+E_{U_{n}I_{m}}\right)}}. \end{array}$$
    After substituting this root into (14) and (15), we can rewrite the end-to-end SINRs at $I_{m}$ and $I_{k}$ as expressed
    (28) $$\begin{array}{cc} \gamma_{e2e,m}^{AF,C1} & =\frac{\upsilon a_{m}\gamma_{B}{\left|{\hat{g}}_{BU_{n}}\right|}^{2}{\left|{\hat{g}}_{U_{n}I_{m}}\right|}^{2}}{\upsilon \gamma_{B}\left(a_{k}{\left|{\hat{g}}_{BU_{n}}\right|}^{2}{\left|{\hat{g}}_{U_{n}I_{m}}\right|}^{2}+{\left|{\hat{g}}_{U_{n}I_{m}}\right|}^{2}E_{BU_{n}}+{\left|{\hat{g}}_{BU_{n}}\right|}^{2}E_{U_{n}I_{m}}+E_{BU_{n}}E_{U_{n}I_{m}}\right)+{\left(B^{C1}\right)}^{2}}, \end{array}$$
    (29) $$\begin{array}{cc} \gamma_{e2e,k}^{AF,C1} & =\frac{\upsilon \gamma_{B}a_{k}A^{C1}{\left|{\hat{g}}_{BU_{n}}\right|}^{2}{\left|{\hat{g}}_{U_{n}I_{k}}\right|}^{2}}{\upsilon \gamma_{B}A^{C1}\left({\left|{\hat{g}}_{U_{n}I_{k}}\right|}^{2}E_{BU_{n}}+{\left|{\hat{g}}_{BU_{n}}\right|}^{2}E_{U_{n}I_{k}}+E_{BU_{n}}E_{U_{n}I_{k}}\right)+\upsilon B^{C1}\left({\left|{\hat{g}}_{U_{n}I_{k}}\right|}^{2}+E_{U_{n}I_{k}}\right)+C^{C1}}, \end{array}$$
    where $A^{C1}$, $B^{C1}$, and $C^{C1}$ are defined as follows:
    (30) $$\begin{array}{cc} A^{C1} & =\sqrt{\upsilon \left({\left|{\hat{g}}_{U_{n}I_{m}}\right|}^{2}+E_{U_{n}I_{m}}\right)}, \end{array}$$
    (31) $$\begin{array}{cc} B^{C1} & =1+A^{C1}, \end{array}$$
    (32) $$\begin{array}{cc} C^{C1} & =A^{C1}B^{C1}. \end{array}$$
  (2) *Case II:* In this case, the APS ratio varies towards achieving the maximum end-to-end SINR for the signal $x_{k}$. Therefore, the APS ratio of the system is
    (33) $$\begin{array}{c} \rho_{*}^{AF,C2}=\rho_{*,k}^{AF}=\frac{\sqrt{\upsilon \left({\left|{\hat{g}}_{U_{n}I_{k}}\right|}^{2}+E_{U_{n}I_{k}}\right)}}{1+\sqrt{\upsilon \left({\left|{\hat{g}}_{U_{n}I_{k}}\right|}^{2}+E_{U_{n}I_{k}}\right)}}. \end{array}$$
    After substituting this root into (14) and (15), we can rewrite the end-to-end SINRs at $I_{m}$ and $I_{k}$ as expressed
    (34) $$\begin{array}{cc} \; & \gamma_{e2e,m}^{AF,C2}=\frac{\upsilon \gamma_{B}a_{m}A^{C2}{\left|{\hat{g}}_{BU_{n}}\right|}^{2}{\left|{\hat{g}}_{U_{n}I_{m}}\right|}^{2}}{\begin{array}{cc} \; & \left[\upsilon \gamma_{B}A^{C2}\left(a_{k}{\left|{\hat{g}}_{BU_{n}}\right|}^{2}{\left|{\hat{g}}_{U_{n}I_{m}}\right|}^{2}+{\left|{\hat{g}}_{U_{n}I_{m}}\right|}^{2}E_{BU_{n}}+{\left|{\hat{g}}_{BU_{n}}\right|}^{2}E_{U_{n}I_{m}}+E_{BU_{n}}E_{U_{n}I_{m}}\right)\right.\\ \; & \left.\;\;\;\;\;\;\;\;\;\;\;\;\;\;\;\;\;\;\;\;\;\;+\upsilon B^{C2}\left({\left|{\hat{g}}_{U_{n}I_{m}}\right|}^{2}+E_{U_{n}I_{m}}\right)+C^{C2}\right] \end{array}}, \end{array}$$
    (35) $$\begin{array}{cc} \; & \gamma_{e2e,k}^{AF,C2}=\frac{\upsilon a_{k}\gamma_{B}{\left|{\hat{g}}_{BU_{n}}\right|}^{2}{\left|{\hat{g}}_{U_{n}I_{k}}\right|}^{2}}{\upsilon \gamma_{B}\left({\left|{\hat{g}}_{U_{n}I_{k}}\right|}^{2}E_{BU_{n}}+{\left|{\hat{g}}_{BU_{n}}\right|}^{2}E_{U_{n}I_{k}}+E_{BU_{n}}E_{U_{n}I_{k}}\right)+{\left(B^{C2}\right)}^{2}}, \end{array}$$
    where $A^{C2}$, $B^{C2}$, and $C^{C2}$ are defined as follows:
    (36) $$\begin{array}{cc} A^{C2} & =\sqrt{\upsilon \left({\left|{\hat{g}}_{U_{n}I_{k}}\right|}^{2}+E_{U_{n}I_{k}}\right)}, \end{array}$$
    (37) $$\begin{array}{cc} B^{C2} & =1+A^{C2}, \end{array}$$
    (38) $$\begin{array}{cc} C^{C2} & =A^{C2}B^{C2}. \end{array}$$

### 3.4. Selection of the Antenna and ID

In this subsection, we demonstrate in detail how to select the best antenna and best ID for both clusters to enhance the quality of communication. The antenna that provides the highest channel gain between itself and the BS in the first phase $\left(B\to U_{n}\right)$ is determined as the best one and is selected as follows [56]:

(39) $$\begin{array}{c} U_{S}=\arg\max_{n=1,\;\ldots ,\;N}\;\left({\left|{\hat{g}}_{BU_{n}}\right|}^{2}\right). \end{array}$$

In the second phase $\left(U_{n}\to I_{i}\right)$, the proposed user selection process is conducted through the signaling and channel state information estimation system. Specifically, a near user and a far user that have the best respective channel conditions will be selected in each transmission slot. Therefore, the BNID $I_{N}$ and the BFID $I_{F}$ can be given as [62]

(40) $$\begin{array}{cc} I_{N} & =\arg\max_{k=1,\;\ldots ,\;K}\;\left({\left|{\hat{g}}_{U_{n}I_{k}}\right|}^{2}\right), \end{array}$$

(41) $$\begin{array}{cc} I_{F} & =\arg\max_{m=1,\;\ldots ,\;M}\;\left({\left|{\hat{g}}_{U_{n}I_{m}}\right|}^{2}\right). \end{array}$$

Note that to save power for the UAV, we use antenna selection to forward information to the IDs instead of using multiple antennas. This means that all antennas maintain established connections at all times. The signals are then combined and presented to the receiver. Depending on the sophistication of the system, the signals can be added directly (equal gain combining) or weighted and added coherently (maximal-ratio combining). Such a system provides the greatest resistance to fading; however, since all of the receive paths must remain energized, it also consumes the most power [63,64].

In this paper, we constrain the fading parameter *m* between links to integer values. Accordingly, the PDF and CDF of ${\left|{\hat{g}}_{XY}\right|}^{2}$ are obtained as follows [46,56]:

(42) $$\begin{array}{cc} f_{{\left|{\hat{g}}_{XY}\right|}^{2}}\left(y\right) & =\frac{Oy^{m_{XY}-1}}{\left(m_{XY}-1\right)!}{\left(\frac{m_{XY}}{{\hat{\Omega }}_{XY}}\right)}^{m_{XY}}e^{-\frac{ym_{XY}}{{\hat{\Omega }}_{XY}}}{\left[1-e^{-\frac{ym_{XY}}{{\hat{\Omega }}_{XY}}}{\sum_{t=0}^{m_{XY}-1}\frac{1}{t!}\left(\frac{ym_{XY}}{{\hat{\Omega }}_{XY}}\right)}^{t}\right]}^{O-1}, \end{array}$$

(43) $$\begin{array}{cc} F_{{\left|{\hat{g}}_{XY}\right|}^{2}}\left(y\right) & =\sum_{ı=0}^{O}\underset{\left(ı\right)}{⋃}{\left(-1\right)}^{ı}\Phi_{ı}^{1}\Phi_{ı}^{2}y^{\bar{ı}}e^{-\frac{ıym_{XY}}{{\hat{\Omega }}_{XY}}}, \end{array}$$

where $XY\in \left\{BU_{S},U_{S}I_{N},U_{S}I_{F}\right\}$, $O\in \left\{N,K,M\right\}$, and $\underset{ı}{⋃}$, $\Phi_{ı}^{1}$, $\Phi_{ı}^{2}$, and $\bar{ı}$ are defined as follows:

(44) $$\begin{array}{cc} \underset{ı}{⋃} & =\sum_{{ı}_{1}=0}^{ı}\sum_{{ı}_{2}=0}^{ı-{ı}_{1}}\ldots \sum_{{ı}_{\left(m_{XY}-1\right)}=0}^{ı-{ı}_{1}-\ldots {ı}_{\left(m_{XY}-2\right)}}, \end{array}$$

(45) $$\begin{array}{cc} \Phi_{ı}^{1} & =\left(\begin{array}{c} O\\ ı \end{array}\right)\left(\begin{array}{c} ı\\ {ı}_{1} \end{array}\right)\left(\begin{array}{c} ı-{ı}_{1}\\ {ı}_{2} \end{array}\right)\ldots \left(\begin{array}{c} ı-{ı}_{1}-\ldots {ı}_{\left(m_{XY}-2\right)}\\ {ı}_{\left(m_{XY}-1\right)} \end{array}\right), \end{array}$$

(46) $$\begin{array}{cc} \Phi_{ı}^{2} & =\prod_{s=0}^{m_{XY}-2}{\left[\frac{1}{s!}{\left(\frac{m_{XY}}{{\hat{\Omega }}_{XY}}\right)}^{s}\right]}^{{ı}_{\left(s+1\right)}}{\left[\frac{1}{\left(m_{XY}-1\right)!}{\left(\frac{m_{XY}}{{\hat{\Omega }}_{XY}}\right)}^{m_{XY}-1}\right]}^{ı-{ı}_{1}-\ldots {ı}_{\left(m_{XY}-1\right)}}, \end{array}$$

(47) $$\begin{array}{cc} \bar{ı} & =\left(m_{XY}-1\right)\left(ı-{ı}_{1}\right)-\left(m_{XY}-2\right){ı}_{2}-\left(m_{XY}-3\right){ı}_{3}\ldots -{ı}_{\left(m_{XY}-1\right)}. \end{array}$$

## 4. Performance Analysis

In this section, we derive closed-form expressions for the OPs in the investigated EH IoT system using a DF/AF UR with NOMA under Nakagami-*m* fading, considering ICSI and the APS ratio. A performance analysis is presented in terms of the OPs for the BFID and BNID; a DF UR is considered in the first subsection, and an AF UR is considered in the second subsection. Finally, we discuss the throughput for each ID in the third subsection.

Following [65,66], the system OP is the probability that the instantaneous SINR falls below a target rate. We let $R_{I_{N}}=R_{I_{F}}=R$, where $R_{I_{N}}$ and $R_{I_{F}}$ (bit/s/Hz) are the target rates for the BNID and BFID, respectively. Thus, the OP $P_{out,I}^{\Xi }$ can be calculated as

(48) $$\begin{array}{cc} P_{out,I}^{\Xi } & =\mathrm{Pr}\left\{\left(1-\alpha \right)\log_{2}\left(1+\gamma_{e2e,I}^{\Xi }\right)<R\right\}\\ \; & =1-\mathrm{Pr}\left\{\gamma_{e2e,I}^{\Xi }>\theta \right\}, \end{array}$$

where $\mathrm{Pr}\left\{.\right\}$ is the probability function, $\Xi \in \left\{DF,AF\right\}$, $I\in \left\{I_{N},I_{F}\right\}$, and $\theta =2^{R/\left(1-\alpha \right)}-1$.

### 4.1. OP Analysis of the DF Scheme

- OP at the BFID

**Lemma** **1.** *The closed-form expression for OP at the BFID is given by*

(49) $$\begin{array}{c} P_{out,I_{F}}^{DF}=F_{{\left|{\hat{g}}_{BU_{S}}\right|}^{2}}\left(\Delta \right)+\frac{\pi \psi }{2Q}\sum_{j=0}^{N-1}\sum_{h=0}^{M}\sum_{q=1}^{Q}\Theta_{\left(j,h\right)}\xi_{1}\vartheta^{m_{BU_{S}}+\bar{j}-1}{\left[-\lambda_{F}\ln\left(\omega_{q}\right)\right]}^{\bar{h}}e^{-\frac{\vartheta \left(j+1\right)m_{BU_{S}}}{{\hat{\Omega }}_{BU_{S}}}}{\omega_{q}}^{\frac{\lambda_{F}hm_{U_{S}I_{F}}}{{\hat{\Omega }}_{U_{S}I_{F}}}}, \end{array}$$

*where $\zeta_{q}=\cos\left(\frac{\pi \left(2q-1\right)}{2Q}\right)$, $\omega_{q}=\frac{\zeta_{q}+1}{2}$, Q is the complexity vs. accuracy trade-off coefficient of the Gauss-Chebyshev quadrature method [67]; and $\vartheta ,\psi ,\lambda_{F},\Theta_{\left(j,h\right)}$, and $\xi_{1}$ are defined as follows:*

(50) $$\begin{array}{cc} \vartheta & =-\frac{1}{\ln\left(\omega_{q}\right)}+\Delta , \end{array}$$

(51) $$\begin{array}{cc} \psi & =\frac{N}{\left(m_{BU_{S}}-1\right)!}{\left(\frac{m_{BU_{S}}}{{\hat{\Omega }}_{BU_{S}}}\right)}^{m_{BU_{S}}}, \end{array}$$

(52) $$\begin{array}{cc} \lambda_{F} & =\frac{\theta \left[-\frac{E_{U_{S}I_{F}}}{\ln\left(\omega_{q}\right)}+\frac{\left(\vartheta -\varpi E_{BU_{S}}\right)}{\upsilon \gamma_{B}\left(\vartheta +E_{BU_{S}}\right)}\right]}{a_{m}-\theta a_{k}}, \end{array}$$

(53) $$\begin{array}{cc} \Theta_{\left(j,h\right)} & =\underset{j}{⋃}\underset{h}{⋃}{\left(-1\right)}^{j+h}\Phi_{j}^{1}\Phi_{j}^{2}\Phi_{h}^{1}\Phi_{h}^{2}, \end{array}$$

(54) $$\begin{array}{cc} \xi_{1} & =\frac{\sqrt{1-{\zeta_{q}}^{2}}}{\omega_{q}\ln^{2}\left(\omega_{q}\right)}. \end{array}$$

**Proof.**  See Appendix A.    □

- OP at the BNID

**Lemma** **2.** *The closed-form expression for the OP at the BNID is given by*

(55) $$\begin{array}{c} P_{out,I_{N}}^{DF}=F_{{\left|{\hat{g}}_{BU_{S}}\right|}^{2}}\left(\Delta \right)+\frac{\pi \psi }{2Q}\sum_{j=0}^{N-1}\sum_{g=0}^{K}\sum_{q=1}^{Q}\Theta_{\left(j,g\right)}\xi_{1}\vartheta^{m_{BU_{S}}+\bar{j}-1}{\left[-\lambda_{N}\ln\left(\omega_{q}\right)\right]}^{\bar{g}}e^{-\frac{\vartheta \left(j+1\right)m_{BU_{S}}}{{\hat{\Omega }}_{BU_{S}}}}{\omega_{q}}^{\frac{\lambda_{N}gm_{U_{S}I_{N}}}{{\hat{\Omega }}_{U_{S}I_{N}}}}, \end{array}$$

*where $\lambda_{N}$ and $\Theta_{\left(j,g\right)}$ are defined as follows:*

(56) $$\begin{array}{cc} \lambda_{N} & =\frac{\theta \left[-\frac{E_{U_{S}I_{N}}}{\ln\left(\omega_{q}\right)}+\frac{\left(\lambda -\varpi E_{BU_{S}}\right)}{\upsilon \gamma_{B}\left(\lambda +E_{BU_{S}}\right)}\right]}{a_{k}}, \end{array}$$

(57) $$\begin{array}{cc} \Theta_{\left(j,g\right)} & =\underset{j}{⋃}\underset{g}{⋃}{\left(-1\right)}^{j+g}\Phi_{j}^{1}\Phi_{j}^{2}\Phi_{g}^{1}\Phi_{g}^{2}. \end{array}$$

**Proof.**  See Appendix B.    □

### 4.2. OP Analysis of the AF Scheme

In this subsection, we analyze the OPs at the BNID and BFID in the two cases APS of the AF scheme presented in Section 3.3.

- For case I:
  (1) OP at the BFID

**Lemma** **3.** *The closed-form expression for the OP at the BFID is given by*

(58) $$\begin{array}{c} P_{out,I_{F}}^{AF,C1}=F_{{\left|{\hat{g}}_{U_{S}I_{F}}\right|}^{2}}\left(\Delta_{F}\right)+\frac{\pi \psi_{F}}{2Q}\sum_{h=0}^{M-1}\sum_{j=0}^{N}\sum_{q=1}^{Q}\Theta_{\left(h,j\right)}\xi_{1}{\varphi_{F}}^{m_{U_{S}I_{F}}+\bar{h}-1}{\left[-\phi_{F}\ln\left(\omega_{q}\right)\right]}^{\bar{j}}e^{-\frac{\left(h+1\right)\varphi_{F}m_{U_{S}I_{F}}}{{\hat{\Omega }}_{U_{S}I_{F}}}}{\omega_{q}}^{\frac{jm_{BU_{S}}\phi_{1,F}}{{\hat{\Omega }}_{BU_{S}}}}, \end{array}$$

*where $\Delta_{F}$, $\varphi_{F},\phi_{1,F},\psi_{F}$, and $\Theta_{\left(h,j\right)}$ are defined as follows:*

(59) $$\begin{array}{cc} \Delta_{F} & =\frac{\theta E_{U_{S}I_{F}}}{\left(a_{m}-\theta a_{k}\right)}, \end{array}$$

(60) $$\begin{array}{cc} \varphi_{F} & =-\frac{1}{\ln\left(\omega_{q}\right)}+\Delta_{F}, \end{array}$$

(61) $$\begin{array}{cc} \phi_{1,F} & =\frac{\Delta_{F}\left[\upsilon \gamma_{B}E_{BU_{S}}\left(\varphi_{F}+E_{U_{S}I_{F}}\right)+{\left(B_{\left(\varphi_{F}\right)}^{C1}\right)}^{2}\right]}{\upsilon \gamma_{B}E_{U_{S}I_{F}}}, \end{array}$$

(62) $$\begin{array}{cc} \psi_{F} & =\frac{M}{\left(m_{U_{S}I_{F}}-1\right)!}{\left(\frac{m_{U_{S}I_{F}}}{{\hat{\Omega }}_{U_{S}I_{F}}}\right)}^{m_{U_{S}I_{F}}}, \end{array}$$

(63) $$\begin{array}{cc} \Theta_{\left(h,j\right)} & =\underset{h}{⋃}\underset{j}{⋃}{\left(-1\right)}^{j+h}\Phi_{h}^{1}\Phi_{h}^{2}\Phi_{j}^{1}\Phi_{j}^{2}. \end{array}$$

**Proof.**  See Appendix C.    □

  (2) OP at the BNID

**Lemma** **4.** *The closed-form expression for the OP at the BFID is given by*

(64) $$\begin{array}{cc} \; & P_{out,I_{N}}^{AF,C1}=1+\frac{\pi^{2}\psi_{1}}{4QW}\sum_{h=0}^{M-1}\sum_{g=0}^{K-1}\sum_{j=1}^{N}\sum_{q=1}^{Q}\sum_{w=1}^{W}\Theta_{\left(h,g,j\right)}\xi_{2}{\left[-\ln^{-1}\left(\omega_{w}\right)\right]}^{m_{U_{S}I_{F}}+\bar{h}-1}\\ \; & \;\;\;\;\;\;\;\;\;\;\times {\varphi_{N}}^{m_{U_{S}I_{N}}+\bar{g}-1}{\left[-\ln\left(\omega_{q}\right)\phi_{1,N}\right]}^{\bar{j}}e^{-\frac{\left(g+1\right)\varphi_{N}m_{U_{S}I_{N}}}{{\hat{\Omega }}_{U_{S}I_{N}}}}{\omega_{w}}^{\frac{\left(h+1\right)m_{U_{S}I_{F}}}{\ln^{2}\left(\omega_{w}\right){\hat{\Omega }}_{U_{S}I_{F}}}}{\omega_{q}}^{\frac{jm_{BU_{S}}\phi_{1,N}}{{\hat{\Omega }}_{BU_{S}}}}, \end{array}$$

*where $\zeta_{w}=\cos\left(\frac{\pi \left(2w-1\right)}{2W}\right)$, $\omega_{w}=\frac{\zeta_{w}+1}{2}$, W is the complexity vs. accuracy trade-off coefficient of the Gauss-Chebyshev quadrature method; and $\Delta_{N}$, $\varphi_{N}$, $\varphi_{1,N}$, $\phi_{1,N}$, $\psi_{1}$, $\Theta_{\left(h,g,j\right)}$, and $\xi_{2}$ are defined as follows:*

(65) $$\begin{array}{cc} \Delta_{N} & =\frac{\theta E_{U_{S}I_{N}}}{a_{k}}, \end{array}$$

(66) $$\begin{array}{cc} \varphi_{N} & =-\frac{1}{\ln\left(\omega_{q}\right)}+\Delta_{N}, \end{array}$$

(67) $$\begin{array}{cc} \varphi_{1,N} & =\upsilon \left(\gamma_{B}A_{\left(-\frac{1}{\ln\left(\omega_{w}\right)}\right)}^{C1}E_{BU_{S}}+B_{\left(-\frac{1}{\ln\left(\omega_{w}\right)}\right)}^{C1}\right), \end{array}$$

(68) $$\begin{array}{cc} \phi_{1,N} & =\frac{\Delta_{N}\left[\varphi_{1,N}\left(\varphi_{N}+E_{U_{S}I_{N}}\right)+C_{\left(-\frac{1}{\ln\left(\omega_{w}\right)}\right)}^{C1}\right]}{\gamma_{B}\upsilon A_{\left(-\frac{1}{\ln\left(\omega_{w}\right)}\right)}^{C1}E_{U_{S}I_{N}}}, \end{array}$$

(69) $$\begin{array}{cc} \psi_{1} & =\frac{\pi^{2}\psi_{1,F}K}{4QW\left(m_{U_{S}I_{N}}-1\right)!}{\left(\frac{m_{U_{S}I_{N}}}{{\hat{\Omega }}_{U_{S}I_{N}}}\right)}^{m_{U_{S}I_{N}}}, \end{array}$$

(70) $$\begin{array}{cc} \Theta_{\left(h,g,j\right)} & =\underset{h}{⋃}\underset{g}{⋃}\underset{j}{⋃}{\left(-1\right)}^{h+j+g}\Phi_{h}^{1}\Phi_{h}^{2}\Phi_{g}^{1}\Phi_{g}^{2}\Phi_{j}^{1}\Phi_{j}^{2}, \end{array}$$

(71) $$\begin{array}{cc} \xi_{2} & =\frac{\sqrt{1-{\zeta_{q}}^{2}}\sqrt{1-{\zeta_{w}}^{2}}}{\omega_{q}\ln^{2}\left(\omega_{q}\right)\omega_{w}\ln^{2}\left(\omega_{w}\right)}. \end{array}$$

**Proof.**  See Appendix D.    □

- For case II:
  (1) OP at the BFID

**Lemma** **5.** *The OP at the BFID is given by*

(72) $$\begin{array}{cc} \; & P_{out,I_{F}}^{AF,C2}=1+\frac{\pi^{2}\psi_{1}}{4QW}\sum_{h=0}^{M-1}\sum_{g=0}^{K-1}\sum_{j=1}^{N}\sum_{q=1}^{Q}\sum_{w=1}^{W}\Theta_{\left(h,g,j\right)}\xi_{2}{\left[-\ln^{-1}\left(\omega_{w}\right)\right]}^{m_{U_{S}I_{N}}+\bar{g}-1}\\ \; & \;\;\;\;\;\;\;\;\;\;\times {\varphi_{F}}^{m_{U_{S}I_{F}}+\bar{h}-1}{\left[-\phi_{2,F}\ln\left(\omega_{q}\right)\right]}^{\bar{j}}e^{-\frac{\left(h+1\right)\varphi_{F}m_{U_{S}I_{F}}}{{\hat{\Omega }}_{U_{S}I_{F}}}}{\omega_{w}}^{\frac{\left(g+1\right)m_{U_{S}I_{N}}}{\ln^{2}\left(\omega_{w}\right){\hat{\Omega }}_{U_{S}I_{N}}}}{\omega_{q}}^{\frac{jm_{BU_{S}}\phi_{2,F}}{{\hat{\Omega }}_{BU_{S}}}}, \end{array}$$

*where $\varphi_{2,F}$ and $\phi_{2,F}$ are defined as follows:*

(73) $$\begin{array}{cc} \varphi_{2,F} & =\upsilon \left(\gamma_{B}A_{\left(-\frac{1}{\ln\left(\omega_{w}\right)}\right)}^{C1}E_{BU_{S}}+B_{\left(-\frac{1}{\ln\left(\omega_{w}\right)}\right)}^{C1}\right), \end{array}$$

(74) $$\begin{array}{cc} \phi_{2,F} & =\frac{\Delta_{N}\left[\varphi_{2,F}\left(\varphi_{N}+E_{U_{S}I_{N}}\right)+C_{\left(-\frac{1}{\ln\left(\omega_{w}\right)}\right)}^{C1}\right]}{\gamma_{B}\upsilon A_{\left(-\frac{1}{\ln\left(\omega_{w}\right)}\right)}^{C1}E_{U_{S}I_{N}}}. \end{array}$$

**Proof.**  See Appendix E.    □

  (2) OP at the BNID

**Lemma** **6.** *The OP at the BNID in case II can be written as*

(75) $$\begin{array}{c} P_{out,I_{N}}^{AF,C2}=F_{{\left|{\hat{g}}_{U_{S}I_{N}}\right|}^{2}}\left(\Delta_{N}\right)+\frac{\pi \psi_{N}}{2Q}\sum_{g=0}^{K-1}\sum_{j=0}^{N}\sum_{q=1}^{Q}\Theta_{\left(g,j\right)}\xi_{1}{\varphi_{N}}^{m_{U_{S}I_{N}}+\bar{g}-1}{\left[-\phi_{2,N}\ln\left(\omega_{q}\right)\right]}^{\bar{j}}e^{-\frac{\left(g+1\right)\varphi_{N}m_{U_{S}I_{N}}}{{\hat{\Omega }}_{U_{S}I_{N}}}}{\omega_{q}}^{\frac{jm_{BU_{S}}\phi_{2,N}}{{\hat{\Omega }}_{BU_{S}}}}, \end{array}$$

*where $\phi_{2,N}$, $\psi_{N}$, and $\Theta_{\left(g,j\right)}$ are defined as follows:*

(76) $$\begin{array}{cc} \phi_{2,N} & =\frac{\Delta_{N}\left[\upsilon \gamma_{B}E_{BU_{S}}\left(\varphi_{N}+E_{U_{S}I_{N}}\right)+{\left(B_{\left(\varphi_{N}\right)}^{C1}\right)}^{2}\right]}{\upsilon \gamma_{B}E_{U_{S}I_{N}}}, \end{array}$$

(77) $$\begin{array}{cc} \psi_{N} & =\frac{K}{\left(m_{U_{S}I_{F}}-1\right)!}{\left(\frac{m_{U_{S}I_{N}}}{{\hat{\Omega }}_{U_{S}I_{N}}}\right)}^{m_{U_{S}I_{N}}}, \end{array}$$

(78) $$\begin{array}{cc} \Theta_{\left(g,j\right)} & =\underset{g}{⋃}\underset{j}{⋃}{\left(-1\right)}^{j+g}\Phi_{g}^{1}\Phi_{g}^{2}\Phi_{j}^{1}\Phi_{j}^{2}. \end{array}$$

**Proof.**  See Appendix F.    □

### 4.3. Throughput Analysis

In this subsection, the throughput for each ID in the delay-limited mode is investigated. Suppose that the source transmits signals to the IDs at a fixed rate; thus, the throughput is mainly determined by the OP [20,48]. The throughput for each ID is given by

(79) $$\begin{array}{c} \tau_{I}=\left(1-\alpha \right)\left(1-P_{out,I}^{\Xi }\right)R. \end{array}$$

The OPs in (49), (55), (58), (64), (72), and (75) are functions of the EH time α [68]. When the value of α is smaller, there is less time for EH and more time for information transmission. Thus, less energy is harvested, and the throughput achieved at the IDs is greater. In contrast, when the value of α is greater, there is more time for EH but less time for information transmission. It is desirable to find the value of α such that the considered system achieves the best performance. However, it is quite challenging to calculate this optimal value of α based on the closed-form expressions obtained above. Therefore, we propose Algorithm 1 to find the nearly optimal EH time for the proposed system [69].

The possible values of α are specified in an array $\left[0\ldots 1\right]$ with *L* elements. We set initial parameters of Δα=0.001, $\alpha^{*}=0$ and $P_{out,I}^{\Xi *}=1$. Next, we update $\alpha^{*}=0$ and $P_{out,I}^{\Xi *}$ through *l* iterations. The iterative process stops when $P_{out,I}^{\Xi *}>P_{out,I}^{\Xi }\left(\Delta \alpha \right)$, and the optimal value of α is found using the formula $\alpha^{*}=\Delta \alpha$ (where Δα is the value when the iterative process stops).
**Algorithm 1** Algorithm for determining the nearly optimal EH time for the system**Input:**$\alpha \in \left[0,\;\ldots ,\;1\right]$**Output:**$\alpha^{*}$ ($\alpha^{*}$ is the optimal point)
1:**function**Loop(α[])2:    $\alpha^{*}$← 0;3:    Δα←0.001;4:    $P_{out,I}^{\Xi *}$← 1;5:    *L*←length(α);6:    **for** l←0 to L−1 **do**7:        **if** $P{}_{out,I_{i}}^{\Xi *}>P_{out,I}^{\Xi }\left(\Delta \alpha \right)$ **then**8:           $\alpha^{*}$←Δα;9:           $P_{out,I}^{\Xi *}\leftarrow P_{out,I}^{\Xi }\left(\Delta \alpha \right)$;10:           Δα←Δα+l;11:        **else**12:           break;13:        **end if**14:    **end for**15:    **return** $\alpha^{*}$;16:**end function**

## 5. Numerical Results

In this section, we present simulation results to validate the OP and throughput analyses of the DF/AF UR-assisted IoT communication network using NOMA with ICSI under Nakagami-*m* fading channels. In particular, we investigate the impacts of the average transmit SNR, the EH time, the number of UR antennas and the number of IDs in each cluster on the OPs and throughput of the BFID and BNID.

Specifically, we consider the following system parameters in all simulations: transmit SNR, $\gamma_{0}$ (dB); distance from *B* to *U*, $d_{BU_{n}}=\sqrt{{\left(h_{U}-h_{B}\right)}^{2}+{d_{BO}}^{2}}$; distance from *U* to far ID, $d_{UI_{m}}=\sqrt{{h_{U}}^{2}+{d_{OI_{m}}}^{2}}$; and distance from *U* to near ID, $d_{UI_{k}}=\sqrt{{h_{U}}^{2}+{d_{OI_{k}}}^{2}}$. The following system parameters are used for both analysis and simulation [18,32,54]: $h_{B}=1$ (m), $d_{BO}\in \left[10,20\right]$ (m), $d_{OI_{i}}\in \left[10,25\right]$ (m), $h_{U}\in \left[10,20\right]$ (m), $m_{BU_{n}}=m_{U_{n}I_{i}}=2$, $\mu_{BU_{n}}=\mu_{U_{n}I_{i}}=0.001$, R∈[0.01,0.5] (bit/s/Hz), α∈[0.1,0.9], η∈[0.1,0.9], $\gamma_{0}\in \left[0,30\right]$ (dB), M=K∈[5,15], and N∈[1,3].

**Figure 3:**
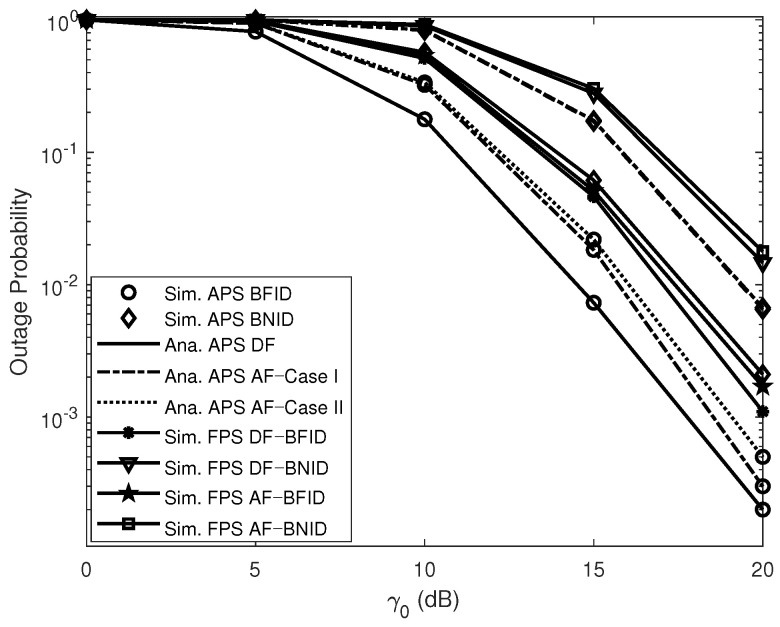
OPs versus $\gamma_{0}$ (dB) DF and AF for BFID and BNID with N=2, M=K=10, α=0.5, η=0.75, and R=0.01 (bit/s/Hz).

Figure 3 shows the impact of the transmit SNR $\gamma_{0}$ (dB) on the OPs. The simulated curves match the analytical curves very closely, illustrating the exactness of the derived expressions. As shown in this figure, the OPs for the BFID and BNID with the DF scheme are lower than those with the AF scheme in all cases. This means that the DF scheme offers better performance than the AF scheme. This finding can be explained as follows. For the DF scheme, the UR needs to confirm that the signals transmitted by the BS have been correctly received (i.e., the received signals must be successfully decoded by the UR) before performing relaying transmission. By contrast, in the AF scheme, the UR merely amplifies the received signals and forwards the information to the IDs. Thus, the reliability for the IDs cannot be improved in the case that the UR cannot decode the received signals successfully. In addition, we compare our investigated system with the corresponding system with a fixed PSR. The results show that our system is superior.

**Figure 4:**
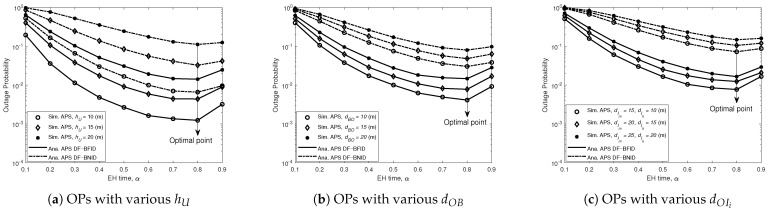
OPs for DF versus EH time (α) for BFID and BNID with various $h_{U}$, $d_{OB}$, and $d_{OI_{i}}$ and with N=2, M=K=10, $\gamma_{0}=20$ (dB), η=0.75, and R=0.01 (bit/s/Hz).

**Figure 5:**
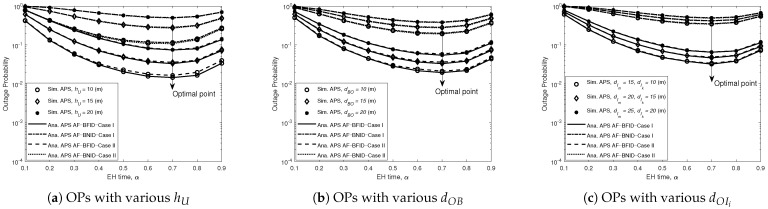
OPs for AF versus EH time (α) BFID and BNID with various $h_{U}$, $d_{OB}$, and $d_{OI_{i}}$ and with N=2, M=K=10, $\gamma_{0}=20$ (dB), η=0.75, and R=0.01 (bit/s/Hz).

Figure 4 and Figure 5 present the effects of the EH time coefficient α on the OPs in different cases of the height of *U* (a), the distance from *B* to *O* (b), and the distance from *U* to the best ID (c), respectively. The value of the EH time coefficient ranges from 0.1 to 0.9, while $\gamma_{0}$ remains at 20 dB. At first glance, it can be seen there is always an optimal value of α for which the value of the OP is minimized. Moreover, the optimal value of α depends on the height of the UR, the distance from *B* to *O*, and the distance from *O* to the ID; specifically, the smaller the $h_{U}$, $d_{BO}$ and $d_{OI_{i}}$ values are, the lower the OP. This is because when the UR is farther from the BS or the ID is farther from the UR, the channel conditions become poorer due to the higher path loss, causing the ID to have difficulty detecting the signal. Another important observation is that the nearly optimal value of α that minimizes the OP is approximately 0.7140 for the AF scheme and 0.7940 for the DF scheme when determined with our proposed algorithm.

**Figure 6:**
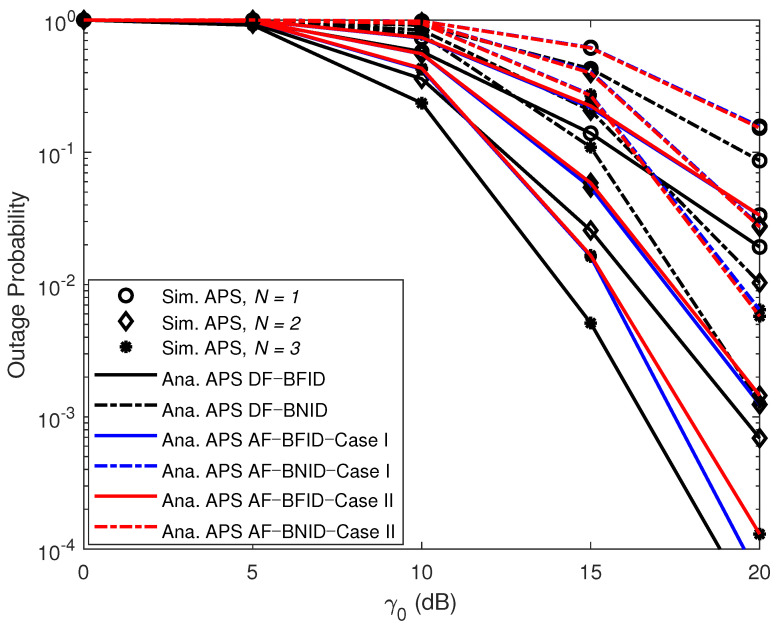
OPs versus $\gamma_{0}$ (dB) DF and AF for BFID and BNID with various numbers of antennas (*N*) and with M=K=10, α=0.4, η=0.75, and R=0.01 (bit/s/Hz).

Figure 6 illustrates the effects of $\gamma_{0}$ (dB) on the OPs for different numbers of antennas (*N*). The results show that the OP of the system improves as the number of antennas increases. Increasing the number of antennas will provide more opportunities for selecting links from *B* to *U*, not only improving the achievable decoding performance but also increasing the amount of energy harvested. Overall, the DF scheme enables better OPs for the system than the AF scheme does.

**Figure 7:**
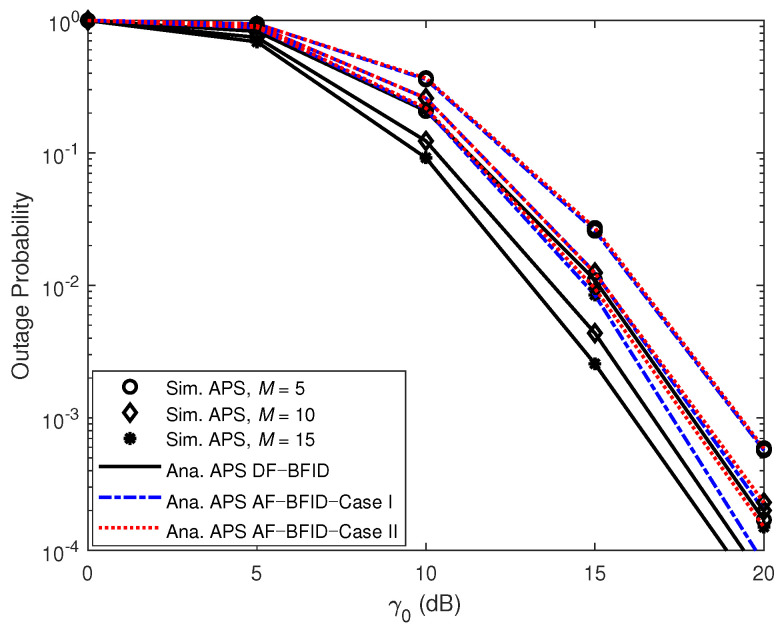
OPs versus $\gamma_{0}$ (dB) DF and AF for BFID and BNID with various numbers of IDs in the far cluster (*M*) and with α=0.4, N=2, η=0.75, and R=0.01 (bit/s/Hz).

**Figure 8:**
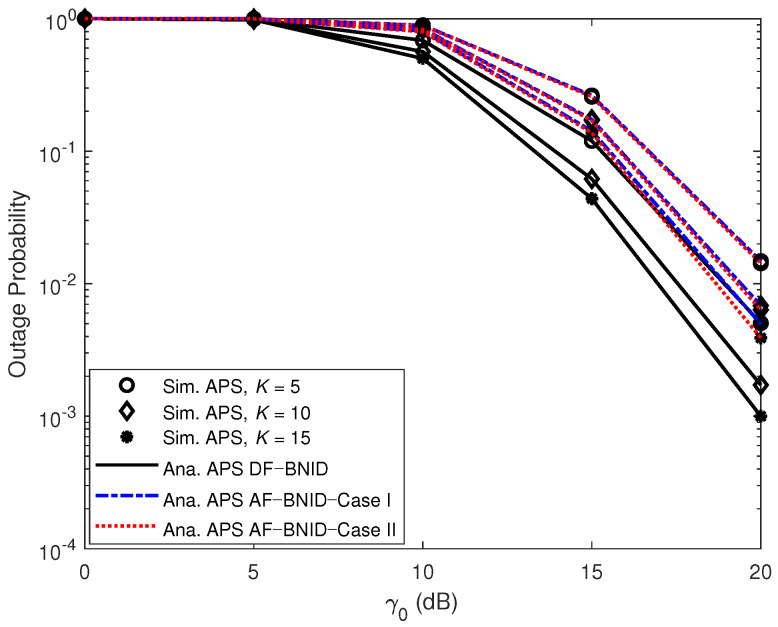
OPs versus $\gamma_{0}$ (dB) DF and AF for BFID and BNID with various numbers of IDs in the near cluster (*K*) and with α=0.4, N=2, η=0.75, and R=0.01 (bit/s/Hz).

Figure 7 and Figure 8 present the impact of $\gamma_{0}$ (dB) on the OPs for different numbers of IDs in the far cluster and the near cluster, respectively. For both clusters, increasing the number of IDs leads to a decrease in the OP. This is because the best ID is chosen based on the channel conditions, and increasing the number of IDs provides a greater variety of possibilities for the best ID.

**Figure 9:**
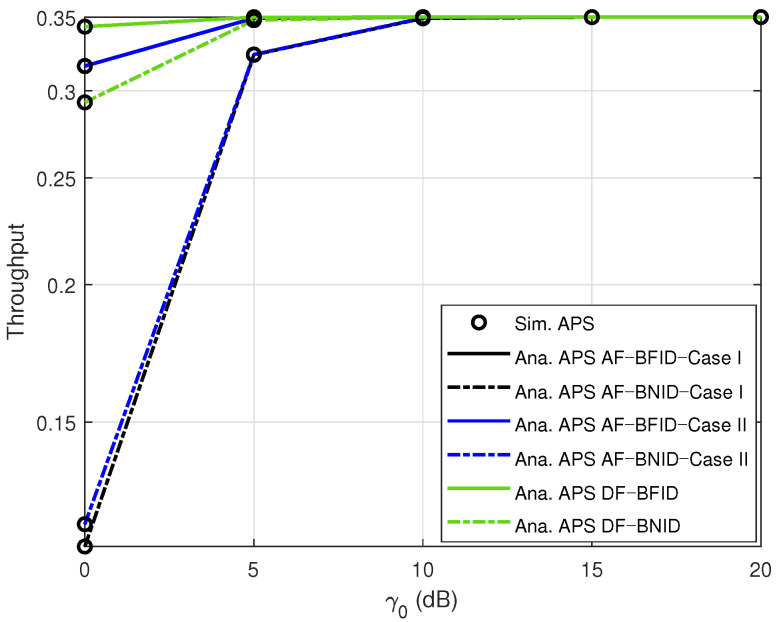
Throughput τ versus $\gamma_{0}$ (dB) DF and AF for BFID and BNID with N=2, M=K=15, α=0.4, η=0.75, and R=0.5 (bit/s/Hz).

Figure 9 shows the impact of $\gamma_{0}$ (dB) on the system throughput. Similar to the OP results, the throughput with the DF scheme is better than that with the AF scheme. Furthermore, in contrast to the OP results, the throughput improves as $\gamma_{0}$ increases within a certain range but then stabilizes when $\gamma_{0}$ is sufficiently large. This is because an ID can obtain the signal more easily when the power of the BS is higher.

**Figure 10:**
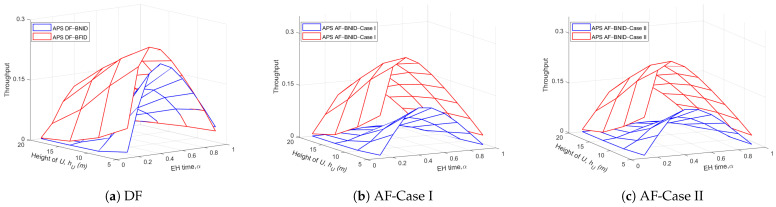
Throughput τ versus α and $h_{U}$ (m) DF and AF for BFID and BNID with N=2, M=K=15, $\gamma_{0}=30$ (dB), η=0.75, and R=0.5 (bit/s/Hz).

**Figure 11:**
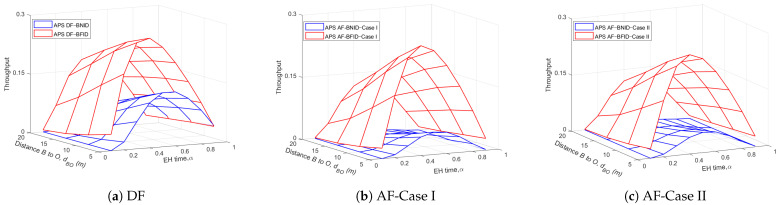
Throughput τ versus α and $d_{BO}$ (m) DF and AF for BFID and BNID with N=2, M=K=15, $\gamma_{0}=30$ (dB), η=0.75, and R=0.5 (bit/s/Hz).

Figure 10 and Figure 11 show the effects of the EH time and the height of the UR or the distance $d_{BO}$ on the throughput for both the DF and AF schemes. The results show that the throughput of the DF scheme is slightly better than that of the AF scheme because the noise at UR is eliminated in the DF scheme, while it is accumulated and amplified in AF scheme. Similar to Figure 4 and Figure 5, we can see that there is always an optimal α value that can maximize the system throughput. In addition, the system throughput improves as the UR height and distance decrease, whereas the throughput decreases with increasing height and distance $d_{OI_{i}}$.

**Figure 12:**
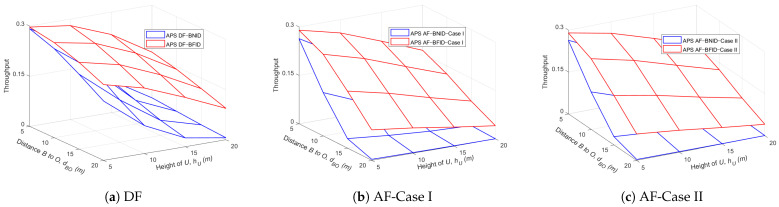
Throughput τ versus $h_{U}$ (m) and $d_{BO}$ (m) DF and AF for BFID and BNID with N=2, M=K=15, $\gamma_{0}=30$ (dB), η=0.75, and R=0.5 (bit/s/Hz).

**Figure 13:**
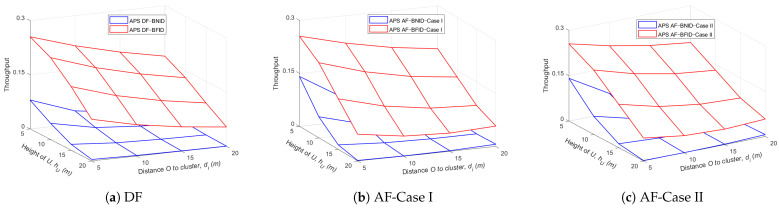
Throughput τ versus $h_{U}$ (m) and $d_{OI_{i}}$ (m) DF and AF for BFID and BNID with N=2, M=K=15, $\gamma_{0}=30$ (dB), η=0.75, and R=0.5 (bit/s/Hz).

Figure 12 and Figure 13 show the effects of the height of the UR and the distance $d_{BO}$ or the distance $d_{OI_{i}}$ on the throughput for both the DF and AF schemes. The results show that the throughput decreases gradually as the UR height and distance increase since the path loss will also increase with an increase in the distance or height, leading to a decrease in the information received at the ID.

## 6. Conclusions

This paper investigated the performance of an RF EH IoT system with a DF/AF UR using downlink NOMA, where the system model consists of a BS, two ID clusters, and a multiantenna UR. A UAV-aided U-TSAPS protocol was adopted to implement EH and information transmission. The system performance of the proposed IoT system under conditions of ICSI and Nakagami-*m* fading was analyzed. Closed-form expressions for the OPs at the BFID and BNID with respect to the APS ratio were derived for performance evaluation, and the throughput for each ID was also evaluated. Accordingly, we proposed an algorithm for finding the optimal EH time that minimizes the OP. The results show that the DF scheme offers better performance than the AF scheme. Moreover, our investigated system with an adaptive PSR outperforms the corresponding fixed-PSR system. In addition, the system performance improves with an increasing number of antennas on the UR and an increasing number of IDs. In future work, we will consider issues related to sensitive and nonlinear EH models [70,71], joint maximum likelihood (ML) decoding [72,73] and imperfect SIC [74,75] for NOMA in IoT systems, including consideration of multihop URs to improve the OP and throughput performance for IoT applications.

## Figures and Tables

**Table 1:** List of notations.

Notation	Description
*B*	The BS
*U*	The energy-limited UR
K,M	The numbers of IDs in the near and far clusters, respectively
*N*	The number of antennas of the UR
$I_{k}$	The *k*-th ID of the near cluster, where k∈{1,…,K}
$I_{m}$	The *m*-th ID of the far cluster, where m∈{1,…,M}
$I_{i}$	The *i*-th ID, where i∈{m,k}
$U_{n}$	The *n*-th antenna of the UR, where n∈{1,…,N}
$h_{U}$	The height of the UR
$h_{B}$	The height of the BS
*O*	The vertical projection point of the UR
$g_{XY}$	The channel coefficient of the X→Y link
$d_{XY}$	The distance of the X→Y link
σ	The path loss exponent
E{.}	The expectation operator
$\Omega_{XY}$	The mean of a RV, where $\Omega_{XY}={d_{XY}}^{-\sigma }$
α	The TSR, where 0≤α≤1
ρ	The PSR, where 0≤ρ≤1
*T*	The length of a time block
$E_{U_{n}}$	The energy harvested at $U_{n}$
$\rho_{*}^{\left(.\right)}$	The APS ratio
$\gamma_{e2e,(.)}^{\left(.\right)}$	The end-to-end SINR
$P_{out,(.)}^{\left(.\right)}$	The OP
*R*	The target rate

## Appendix A. Proof of Lemma 1

Following [57], the OP at the BFID can be written as

(A1) $$\begin{array}{c} P_{out,I_{F}}^{DF}=1-\mathrm{Pr}\left\{P_{U_{S}}>0,\gamma_{e2e,I_{F}}^{DF}>\theta \right\}. \end{array}$$

By substituting (21) and (22) into (A1), we can rewrite the OP at the BFID as

(A2) $$\begin{array}{c} P_{out,I_{F}}^{DF}=1-\mathrm{Pr}\left\{{\left|{\hat{g}}_{BU_{S}}\right|}^{2}>\Delta ,{\left|{\hat{g}}_{U_{S}I_{F}}\right|}^{2}>{\bar{\lambda }}_{F}^{\left({\left|{\hat{g}}_{BU_{S}}\right|}^{2}\right)}\right\}, \end{array}$$

where ${\bar{\lambda }}_{F}^{\left({\left|{\hat{g}}_{BU_{S}}\right|}^{2}\right)}=\frac{\theta \left[\left({\left|{\hat{g}}_{BU_{S}}\right|}^{2}-\Delta \right)E_{U_{S}I_{F}}+\frac{{\left|{\hat{g}}_{BU_{S}}\right|}^{2}-\varpi E_{BU_{S}}}{\upsilon \gamma_{B}\left({\left|{\hat{g}}_{BU_{S}}\right|}^{2}+E_{BU_{S}}\right)}\right]}{\left(a_{m}-\theta a_{k}\right)\left({\left|{\hat{g}}_{BU_{S}}\right|}^{2}-\Delta \right)}$.

Based on (A2), the OP at the BFID can be rewritten as

(A3) $$\begin{array}{cc} P_{out,I_{F}}^{DF} & =1-\int_{\Delta }^{\infty }f_{{\left|{\hat{g}}_{BU_{S}}\right|}^{2}}\left(y\right)\left[1-F_{{\left|{\hat{g}}_{U_{S}I_{F}}\right|}^{2}}\left({\bar{\lambda }}_{F}^{\left(y\right)}\right)\right]dy\\ \; & =\int_{0}^{\Delta }f_{{\left|{\hat{g}}_{BU_{S}}\right|}^{2}}\left(y\right)dy+\int_{\Delta }^{\infty }f_{{\left|{\hat{g}}_{BU_{S}}\right|}^{2}}\left(y\right)F_{{\left|{\hat{g}}_{U_{S}I_{F}}\right|}^{2}}\left({\bar{\lambda }}_{F}^{\left(y\right)}\right)dy. \end{array}$$

By combining the PDF in (42) and the CDF in (43) into (A3), we can rewrite the OP at the BFID as

(A4) $$\begin{array}{cc} \; & P_{out,I_{{}_{F}}}^{DF}=F_{{\left|{\hat{g}}_{BU_{S}}\right|}^{2}}\left(\Delta \right)+\psi \sum_{j=0}^{N-1}\sum_{h=0}^{M}\Theta_{\left(j,h\right)}\underset{\Psi_{I_{F}}^{DF}}{\underbrace{\int_{\Delta }^{\infty }y^{m_{BU_{S}}+\bar{j}-1}{\left[{\bar{\lambda }}_{F}^{\left(y\right)}\right]}^{\bar{h}}e^{-\frac{\left(j+1\right)ym_{BU_{S}}}{{\hat{\Omega }}_{BU_{S}}}-\frac{{\bar{\lambda }}_{F}^{\left(y\right)}hm_{U_{S}I_{F}}}{{\hat{\Omega }}_{U_{S}I_{F}}}}dy}}. \end{array}$$

Let z=y−Δ; then, $\Psi_{I_{F}}^{DF}$ can be written as

(A5) $$\begin{array}{cc} \Psi_{I_{F}}^{DF} & =\int_{0}^{\infty }{\left(z+\Delta \right)}^{m_{BU_{S}}+\bar{j}-1}{\left[{\bar{\lambda }}_{F}^{\left(z+\Delta \right)}\right]}^{\bar{h}}e^{-\frac{\left(j+1\right)\left(z+\Delta \right)m_{BU_{S}}}{{\hat{\Omega }}_{BU_{S}}}-\frac{{\bar{\lambda }}_{F}^{\left(z+\Delta \right)}hm_{U_{S}I_{F}}}{{\hat{\Omega }}_{U_{S}I_{F}}}}dz. \end{array}$$

Let $t=e^{-\frac{1}{z}}$ and $\Psi_{I_{F}}^{DF}$ in (A5) can be rewritten as

(A6) $$\begin{array}{cc} \Psi_{I_{F}}^{DF} & =\int_{0}^{1}\frac{{\left(-\ln^{-1}\left(t\right)+\Delta \right)}^{m_{BU_{S}}+\bar{j}-1}}{t\ln^{2}\left(t\right)}{\left[{\bar{\lambda }}_{F}^{\left(-\ln^{-1}\left(t\right)\right)}\right]}^{\bar{h}}e^{-\frac{\left(j+1\right)\left(-\ln^{-1}\left(t\right)+\Delta \right)m_{BU_{S}}}{{\hat{\Omega }}_{BU_{S}}}}t^{\frac{-\ln^{-1}\left(t\right){\bar{\lambda }}_{F}^{\left(-\ln^{-1}\left(t\right)\right)}hm_{U_{S}I_{F}}}{{\hat{\Omega }}_{U_{S}I_{F}}}}dt. \end{array}$$

Next, the Gauss-Chebyshev quadrature method [67] is applied to solve the integral in (A6), $\Psi_{I_{F}}^{DF}$ can be expressed as

(A7) $$\begin{array}{cc} \Psi_{I_{F}}^{DF} & =\frac{\pi }{2Q}\sum_{q=1}^{Q}\xi_{1}\vartheta^{m_{BU_{S}}+\bar{j}-1}{\left[-\lambda_{F}\ln\left(\omega_{q}\right)\right]}^{\bar{h}}e^{-\frac{\vartheta \left(j+1\right)m_{BU_{S}}}{{\hat{\Omega }}_{BU_{S}}}}{\omega_{q}}^{\frac{\lambda_{F}hm_{U_{S}I_{F}}}{{\hat{\Omega }}_{U_{S}I_{F}}}}, \end{array}$$

where $\zeta_{q}=\cos\left(\frac{\pi \left(2q-1\right)}{2Q}\right)$, $\omega_{q}=\frac{\zeta_{q}+1}{2}$, and *Q* is the complexity vs. accuracy trade-off coefficient of the Gauss-Chebyshev quadrature method. By substituting (A7) into (A4), the proof of Lemma 1 is completed.

## Appendix B. Proof of Lemma 2

Similar to (A1), the OP at the BNID is defined as follows:

(A8) $$\begin{array}{c} P_{out,I_{N}}^{DF}=1-\mathrm{Pr}\left\{P_{U_{S}}>0,\gamma_{e2e,I_{N}}^{DF}>\theta_{I_{N}}^{DF}\right\}. \end{array}$$

By substituting (21) and (23) into (A8), we can rewrite the OP at the BNID as

(A9) $$\begin{array}{c} P_{out,I_{}}^{DF}=1-\mathrm{Pr}\left\{{\left|{\hat{g}}_{BU_{S}}\right|}^{2}>\Delta ,{\left|{\hat{g}}_{U_{S}I_{N}}\right|}^{2}>{\bar{\lambda }}_{N}^{\left({\left|{\hat{g}}_{BU_{S}}\right|}^{2}\right)}\right\}, \end{array}$$

where ${\bar{\lambda }}_{N}^{\left({\left|{\hat{g}}_{BU_{S}}\right|}^{2}\right)}=\frac{\theta \left[\left({\left|{\hat{g}}_{BU_{S}}\right|}^{2}-\Delta \right)E_{U_{S}I_{N}}+\frac{{\left|{\hat{g}}_{BU_{S}}\right|}^{2}-\varpi E_{BU_{S}}}{\upsilon \gamma_{B}\left({\left|{\hat{g}}_{BU_{S}}\right|}^{2}+E_{BU_{S}}\right)}\right]}{a_{k}\left({\left|{\hat{g}}_{BU_{S}}\right|}^{2}-\Delta \right)}$.

By again using the PDF and CDF in (42) and (43), we can rewrite the OP at the BNID as

(A10) $$\begin{array}{cc} \; & P_{out,I_{N}}^{DF}=F_{{\left|{\hat{g}}_{BU_{S}}\right|}^{2}}\left(\Delta \right)+\psi \sum_{j=0}^{N-1}\sum_{g=0}^{K}\Theta_{\left(j,g\right)}\underset{\Psi_{I_{N}}^{DF}}{\underbrace{\int_{\Delta }^{\infty }y^{m_{BU_{S}}+\bar{j}-1}{\left[{\bar{\lambda }}_{N}^{\left(y\right)}\right]}^{\bar{g}}e^{-\frac{\left(j+1\right)ym_{BU_{S}}}{{\hat{\Omega }}_{BU_{S}}}-\frac{{\bar{\lambda }}_{N}^{\left(y\right)}gm_{U_{S}I_{N}}}{{\hat{\Omega }}_{U_{S}I_{N}}}}dy}}. \end{array}$$

From (A10), the analysis of $\Psi_{I_{N}}^{DF}$ follows steps similar to those in Appendix A. Finally, the closed-form expression for the OP at the BNID is given in Lemma 2.

## Appendix C. Proof of Lemma 3

From (48), the OP at the BFID can be written as

(A11) $$\begin{array}{c} P_{out,I_{F}}^{AF,C1}=1-\mathrm{Pr}\left\{\gamma_{e2e,I_{F}}^{AF,C1}>\theta \right\}. \end{array}$$

By substituting (28) into (A11), we can rewrite the OP at the BFID as

(A12) $$\begin{array}{c} P_{out,I_{F}}^{AF,C1}=1-\mathrm{Pr}\left\{{\left|{\hat{g}}_{U_{S}I_{F}}\right|}^{2}>\Delta_{F},{\left|{\hat{g}}_{BU_{S}}\right|}^{2}>{\bar{\phi }}_{1{,}_{F}}^{\left({\left|{\hat{g}}_{U_{S}I_{F}}\right|}^{2}\right)}\right\}, \end{array}$$

where ${\bar{\phi }}_{1{,}_{F}}^{\left({\left|{\hat{g}}_{U_{S}I_{F}}\right|}^{2}\right)}=\frac{\Delta_{F}\left[\upsilon \gamma_{B}E_{BU_{S}}\left({\left|{\hat{g}}_{U_{S}I_{F}}\right|}^{2}+E_{U_{S}I_{F}}\right)+B{{}_{\left({\left|{\hat{g}}_{U_{S}I_{F}}\right|}^{2}\right)}^{C1}}^{2}\right]}{\upsilon \gamma_{B}E_{U_{S}I_{F}}\left({\left|{\hat{g}}_{U_{S}I_{F}}\right|}^{2}-\Delta_{F}\right)}$.

Equation (A12) is obtained on the condition that $a_{m}>\theta a_{k}$ [55]. Using the conditional probability property with respect to *y*, $P_{out,I_{F}}^{AF,C1}$ for the BFID can be rewritten as

(A13) $$\begin{array}{cc} P_{out,I_{F}}^{AF,C1} & =\int_{0}^{\Delta_{F}}f_{{\left|{\hat{g}}_{U_{S}I_{F}}\right|}^{2}}\left(y\right)dy+\int_{\Delta_{F}}^{\infty }f_{{\left|{\hat{g}}_{U_{S}I_{F}}\right|}^{2}}\left(y\right)F_{{\left|{\hat{g}}_{BU_{S}}\right|}^{2}}\left({\bar{\phi }}_{1{,}_{F}}^{\left(y\right)}\right)dy, \end{array}$$

By plugging the PDF in (42) and the CDF in (43) into (A13), we can rewrite the OP at the BFID as

(A14) $$\begin{array}{cc} P_{out,I_{F}}^{AF,C1} & =F_{{\left|{\hat{g}}_{U_{S}I_{F}}\right|}^{2}}\left(\Delta_{F}\right)+\psi_{F}\sum_{h=0}^{M-1}\sum_{j=1}^{N}\Theta_{\left(h,j\right)}\underset{\Psi_{I_{F}}^{AF,C1}}{\underbrace{\int_{\Delta_{F}}^{\infty }y^{m_{U_{S}I_{F}}+\bar{h}-1}{\left[{\bar{\phi }}_{1{,}_{F}}^{\left(y\right)}\right]}^{\bar{j}}e^{-\frac{\left(h+1\right)ym_{U_{S}I_{F}}}{{\hat{\Omega }}_{U_{S}I_{F}}}-\frac{{\bar{\phi }}_{1{,}_{F}}^{\left(y\right)}jm_{BU_{S}}}{{\hat{\Omega }}_{BU_{S}}}}dy}}. \end{array}$$

From (A14), the analysis of $\Psi_{I_{F}}^{AF,C1}$ follows steps similar to those in Appendix A. The proof of Lemma 3 is completed.

## Appendix D. Proof of Lemma 4

Similar to (A11), the OP at the BNID can be written as

(A15) $$\begin{array}{c} P_{out,I_{N}}^{AF,C1}=1-\mathrm{Pr}\left\{\gamma_{e2e,I_{N}}^{AF,C1}>\theta \right\}. \end{array}$$

By substituting the end-to-end SINR at the BNID given in (29) into (A15) and letting ${\left|{\hat{g}}_{U_{S}I_{F}}\right|}^{2}=x$ and ${\left|{\hat{g}}_{U_{S}I_{N}}\right|}^{2}=y$, $P_{out,I_{N}}^{AF,C1}$ can rewrite as

(A16) $$\begin{array}{c} P_{out,I_{N}}^{AF,C1}=1-\int_{0}^{\infty }\mathrm{Pr}\left\{y>\Delta_{N},{\left|{\hat{g}}_{BU_{S}}\right|}^{2}>{\bar{\phi }}_{1,N}^{\left(x,y\right)}\right\}f_{{\left|{\hat{g}}_{U_{S}I_{F}}\right|}^{2}}\left(x\right)dx, \end{array}$$

where ${\bar{\phi }}_{1,N}^{\left(x,y\right)}=\frac{\Delta_{N}\left[\upsilon \left(\gamma_{B}A_{\left(x\right)}^{C1}E_{BU_{n}}+B_{\left(x\right)}^{C1}\right)\left(y+E_{U_{S}I_{N}}\right)+C_{\left(x\right)}^{C1}\right]}{\gamma_{B}\upsilon A_{\left(x\right)}^{C1}E_{U_{S}I_{N}}\left(y-\Delta_{N}\right)}$.

Using the conditional probability property with respect to *y*, the OP at the BFID can be rewritten as

(A17) $$\begin{array}{c} P_{out,I_{N}}^{AF,C1}=1-\underset{\Psi_{2,I_{N}}^{AF,C1}}{\underbrace{\int_{0}^{\infty }\underset{\Psi_{1,I_{N}}^{AF,C1}}{\underbrace{\int_{\Delta_{N}}^{\infty }\left[1-F_{{\left|{\hat{g}}_{BU_{S}}\right|}^{2}}\left({\bar{\phi }}_{1,N}^{\left(x,y\right)}\right)\right]f_{{\left|{\hat{g}}_{U_{S}I_{N}}\right|}^{2}}\left(y\right)dy}}f_{{\left|{\hat{g}}_{U_{S}I_{F}}\right|}^{2}}\left(x\right)dx}}. \end{array}$$

By substituting (42) and (43) into (A17). After some manipulation similar to Appendix A, $\Psi_{1,I_{N}}^{AF,C1}$ can be rewritten as

(A18) $$\begin{array}{cc} \Psi_{1,I_{N}}^{AF,C1} & =-\frac{\pi \psi_{N}}{2Q}\sum_{g=0}^{K-1}\sum_{j=1}^{N}\sum_{q=1}^{Q}\Theta_{\left(g,j\right)}\xi_{1}{\varphi_{N}}^{m_{U_{S}I_{N}}+\bar{g}-1}{\left[{\bar{\phi }}_{1,N}^{\left(x,-\ln^{-1}\left(\omega_{q}\right)\right)}\right]}^{\bar{j}}\\ \; & \;\;\;\;\;\;\;\;\times {\omega_{q}}^{\frac{-\ln^{-1}\left(\omega_{q}\right){\bar{\phi }}_{1,N}^{\left(x,-\ln^{-1}\left(\omega_{q}\right)\right)}jm_{BU_{S}}}{{\hat{\Omega }}_{BU_{S}}}}e^{-\frac{\varphi_{N}\left(g+1\right)m_{U_{S}I_{N}}}{{\hat{\Omega }}_{U_{S}I_{N}}}}. \end{array}$$

Next, we substitute $\Psi_{1,I_{N}}^{AF,C1}$ in (A18) and the PDF in (42) into $\Psi_{2,I_{N}}^{AF,C1}$ in (A17); finally, let $u=e^{-\frac{1}{x}}$. By again applying the Gauss-Chebyshev quadrature method to solve the integral, $\Psi_{2,I_{N}}^{AF,C1}$ can be rewritten as

(A19) $$\begin{array}{cc} \Psi_{2,I_{N}}^{AF,C1} & =-\frac{\pi \psi_{1}}{2Q}\sum_{h=0}^{M-1}\sum_{g=0}^{K-1}\sum_{j=1}^{N}\sum_{q=1}^{Q}\Theta_{\left(h,g,j\right)}\xi_{1}{\varphi_{N}}^{m_{U_{S}I_{N}}+\bar{g}-1}e^{-\frac{\varphi_{N}\left(g+1\right)m_{U_{S}I_{N}}}{{\hat{\Omega }}_{U_{S}I_{N}}}}\\ \; & \;\;\;\;\;\;\;\times \int_{0}^{\infty }x^{m_{U_{S}I_{F}}+\bar{h}-1}{\left[{\bar{\phi }}_{1,N}^{\left(x\right)}\right]}^{\bar{j}}e^{-\frac{x\left(h+1\right)m_{U_{S}I_{F}}}{{\hat{\Omega }}_{U_{S}I_{F}}}}{\omega_{q}}^{\frac{-\ln^{-1}\left(\omega_{q}\right){\bar{\phi }}_{1,N}^{\left(x\right)}jm_{BU_{S}}}{{\hat{\Omega }}_{BU_{S}}}}dx\\ \; & =-\frac{\pi^{2}\psi_{1}}{4QW}\sum_{h=0}^{M-1}\sum_{g=1}^{K}\sum_{j=0}^{N-1}\sum_{q=1}^{Q}\sum_{w=1}^{W}\Theta_{\left(h,g,j\right)}\xi_{2}{\left[-\ln^{-1}\left(\omega_{w}\right)\right]}^{m_{U_{S}I_{F}}+\bar{h}-1}{\varphi_{N}}^{m_{U_{S}I_{N}}+\bar{g}-1}\\ \; & \;\;\;\;\;\;\;\times {\left[-\phi_{1,N}\ln\left(\omega_{q}\right)\right]}^{\bar{j}}e^{-\frac{\varphi_{N}\left(g+1\right)m_{U_{S}I_{N}}}{{\hat{\Omega }}_{U_{S}I_{N}}}}{\omega_{w}}^{\frac{\left(h+1\right)m_{U_{S}I_{F}}}{\ln^{2}\left(\omega_{w}\right){\hat{\Omega }}_{U_{S}I_{F}}}}{\omega_{q}}^{\frac{\phi_{1,N}jm_{BU_{S}}}{{\hat{\Omega }}_{BU_{S}}}}. \end{array}$$

By substituting (A19) into (A17), the proof is completed.

## Appendix E. Proof of Lemma 5

Similar to (A11), the OP at the BFID can be expressed as

(A20) $$\begin{array}{c} P_{out,I_{F}}^{AF,C2}=1-\mathrm{Pr}\left\{\gamma_{e2e,I_{F}}^{AF,C2}>\theta \right\}. \end{array}$$

By substituting (34) into (A20) and letting ${\left|{\hat{g}}_{U_{S}I_{N}}\right|}^{2}=x$ and ${\left|{\hat{g}}_{U_{S}I_{F}}\right|}^{2}=y$, similar to (A17), $P_{out,I_{F}}^{AF,C2}$ can be rewritten as

(A21) $$\begin{array}{cc} P_{out,I_{F}}^{AF,C2} & =1-\int_{0}^{\infty }\mathrm{Pr}\left\{y>\Delta_{F},\right.\left.{\left|{\hat{g}}_{BU_{S}}\right|}^{2}>{\bar{\phi }}_{2,F}^{\left(x,y\right)}\right\}f_{{\left|{\hat{g}}_{U_{S}I_{N}}\right|}^{2}}\left(x\right)dx\\ \; & =1-\underset{\Psi_{2,I_{F}}^{AF,C2}}{\underbrace{\int_{0}^{\infty }\underset{\Psi_{1,I_{F}}^{AF,C2}}{\underbrace{\int_{\Delta_{F}}^{\infty }\left[1-F_{{\left|{\hat{g}}_{BU_{S}}\right|}^{2}}\left({\bar{\phi }}_{2,F}^{\left(x,y\right)}\right)\right]f_{{\left|{\hat{g}}_{U_{S}I_{F}}\right|}^{2}}\left(y\right)dy}}f_{{\left|{\hat{g}}_{U_{S}I_{N}}\right|}^{2}}\left(x\right)dx}}, \end{array}$$

where ${\bar{\phi }}_{2,F}^{\left(x,y\right)}=\frac{\Delta_{F}\left[\upsilon \left(\gamma_{B}A_{\left(x\right)}^{C2}E_{BU_{S}}+B_{\left(x\right)}^{C2}\right)\left(y+E_{U_{S}I_{F}}\right)+C_{\left(x\right)}^{C2}\right]}{\upsilon \gamma_{B}A_{\left(x\right)}^{C2}\left(y-\Delta_{F}\right)}$.

From (A21), $\Psi_{1,I_{F}}^{AF,C2}$ and $\Psi_{2,I_{F}}^{AF,C2}$ are derived by using the steps shown in Appendix D.

## Appendix F. Proof of Lemma 6

Similar to (A15), the OP at the BNID in case II is defined as follows:

(A22) $$\begin{array}{c} P_{out,I_{N}}^{AF,C2}=1-\mathrm{Pr}\left\{\gamma_{e2e,I_{N}}^{AF,C2}>\theta \right\}. \end{array}$$

By substituting (35) into (A22), $P_{out,I_{N}}^{AF,C2}$ can be rewritten as

(A23) $$\begin{array}{cc} P_{out,I_{N}}^{AF,C2} & =1-\mathrm{Pr}\left\{{\left|{\hat{g}}_{U_{S}I_{N}}\right|}^{2}>\Delta_{N},{\left|{\hat{g}}_{BU_{S}}\right|}^{2}>{\bar{\phi }}_{2,N}^{\left({\left|{\hat{g}}_{U_{S}I_{N}}\right|}^{2}\right)}\right\}, \end{array}$$

where ${\bar{\phi }}_{2,N}^{\left({\left|{\hat{g}}_{U_{S}I_{N}}\right|}^{2}\right)}=\frac{\Delta_{N}\left[\upsilon \gamma_{B}E_{BU_{S}}\left({\left|{\hat{g}}_{U_{S}I_{N}}\right|}^{2}+E_{U_{S}I_{N}}\right)+B{{}_{\left({\left|{\hat{g}}_{U_{S}I_{N}}\right|}^{2}\right)}^{C2}}^{2}\right]}{\upsilon \gamma_{B}E_{U_{S}I_{N}}\left({\left|{\hat{g}}_{U_{S}I_{N}}\right|}^{2}-\Delta_{N}\right)}$.

Using the conditional probability property with respect to *y* and after some manipulations, we obtain the expression for the OP given by

(A24) $$\begin{array}{cc} P_{out,I_{N}}^{AF,C2} & =\int_{0}^{\Delta_{N}}f_{{\left|{\hat{g}}_{U_{S}I_{N}}\right|}^{2}}\left(y\right)dy+\int_{\Delta_{N}}^{\infty }f_{{\left|{\hat{g}}_{U_{S}I_{N}}\right|}^{2}}\left(y\right)F_{{\left|{\hat{g}}_{BU_{S}}\right|}^{2}}\left({\bar{\phi }}_{2,N}^{\left(y\right)}\right)dy\\ \; & =F_{{\left|{\hat{g}}_{U_{S}I_{N}}\right|}^{2}}\left(\Delta_{N}\right)+\psi_{N}\sum_{g=0}^{K-1}\sum_{j=1}^{N}\Theta_{\left(g,j\right)}\underset{\Psi_{I_{N}}^{AF,C2}}{\underbrace{\int_{\Delta_{N}}^{\infty }y^{m_{U_{S}I_{F}}+\bar{g}-1}{\left[{\bar{\phi }}_{2,N}^{\left(y\right)}\right]}^{\bar{j}}e^{-\frac{\left(g+1\right)ym_{U_{S}I_{F}}}{{\hat{\Omega }}_{U_{S}I_{F}}}-\frac{{\bar{\phi }}_{2,N}^{\left(y\right)}gm_{BU_{S}}}{{\hat{\Omega }}_{BU_{S}}}}dy}}. \end{array}$$

From (A24), $\Psi_{I_{N}}^{AF,C2}$ is derived by using the steps shown in Appendix C.
